# The small molecule inhibitor YK-4-279 disrupts mitotic progression of neuroblastoma cells, overcomes drug resistance and synergizes with inhibitors of mitosis

**DOI:** 10.1016/j.canlet.2017.05.027

**Published:** 2017-09-10

**Authors:** Madhu Kollareddy, Alice Sherrard, Ji Hyun Park, Marianna Szemes, Kelli Gallacher, Zsombor Melegh, Sebastian Oltean, Martin Michaelis, Jindrich Cinatl, Abderrahmane Kaidi, Karim Malik

**Affiliations:** a Cancer Epigenetics Laboratory, School of Cellular and Molecular Medicine, University of Bristol, Bristol, UK; b Nuclear Dynamics Laboratory, School of Cellular and Molecular Medicine, University of Bristol, Bristol, UK; c School of Physiology and Pharmacology, University of Bristol, Bristol, UK; d Centre for Molecular Processing and School of Biosciences, University of Kent, Canterbury, UK; e Institut für Medizinische Virologie, Klinikum der Goethe-Universität, Frankfurt am Main, Germany

**Keywords:** Neuroblastoma, Chemotherapy, YK-4-279, Mitosis, Drug resistance/synergy, ALK, Anaplastic Lymphoma kinase, EGF, Epidermal growth factor, ERK, extracellular signal-regulated kinases, GFP, green fluorescent protein, kDa, kilodaltons, MAPK, mitogen-activated protein kinase, MEK, Mitogen-activated protein kinase kinase, MTT, 3-(4,5-Dimethylthiazol-2-Yl)-2,5-Diphenyltetrazolium Bromide, PBS, Phosphate-buffered saline, pHH3, phospho-histone H3 (ser10), RNase A, ribonuclease A, QVD, quinolyl-valyl-*O*-methylaspartyl-(-2,6-difluorophenoxy)- methyl ketone, SDS-PAGE, sodium-dodecyl sulphate-polyacrylamide gel electrophoresis, TERT, Telomerase reverse transcriptase

## Abstract

Neuroblastoma is a biologically and clinically heterogeneous pediatric malignancy that includes a high-risk subset for which new therapeutic agents are urgently required. As well as *MYCN* amplification, activating point mutations of *ALK* and *NRAS* are associated with high-risk and relapsing neuroblastoma. As both ALK and RAS signal through the MEK/ERK pathway, we sought to evaluate two previously reported inhibitors of ETS-related transcription factors, which are transcriptional mediators of the Ras-MEK/ERK pathway in other cancers. Here we show that YK-4-279 suppressed growth and triggered apoptosis in nine neuroblastoma cell lines, while BRD32048, another ETV1 inhibitor, was ineffective. These results suggest that YK-4-279 acts independently of ETS-related transcription factors. Further analysis reveals that YK-4-279 induces mitotic arrest in prometaphase, resulting in subsequent cell death. Mechanistically, we show that YK-4-279 inhibits the formation of kinetochore microtubules, with treated cells showing a broad range of abnormalities including multipolar, fragmented and unseparated spindles, together leading to disrupted progression through mitosis. Notably, YK-4-279 does not affect microtubule acetylation, unlike the conventional mitotic poisons paclitaxel and vincristine. Consistent with this, we demonstrate that YK-4-279 overcomes vincristine-induced resistance in two neuroblastoma cell-line models. Furthermore, combinations of YK-4-279 with vincristine, paclitaxel or the Aurora kinase A inhibitor MLN8237/Alisertib show strong synergy, particularly at low doses. Thus, YK-4-279 could potentially be used as a single-agent or in combination therapies for the treatment of high-risk and relapsing neuroblastoma, as well as other cancers.

## Highlights

• The small molecule inhibitor YK-4-279 can trigger p53-independent apoptosis in neuroblastoma cell lines.
• YK-4-279 induces mitotic arrest with multipolar, fragmented and unseparated spindles.
• YK-4-279 acts differently from paclitaxel and vincristine.
• YK-4-279 can overcome resistance to vincristine and synergize with other inhibitors of mitosis.

## Introduction

The clinical heterogeneity of neuroblastoma is reflected in the complex molecular aetiology of this frequently lethal childhood cancer. Amongst high-risk tumours, which comprise about half of all neuroblastomas, approximately 40% are dependent on *MYCN* gene amplification (MNA). Despite extensive genome and transcriptome sequencing analyses, oncogenic mutations in neuroblastoma are comparatively rare compared to other cancers [1], [2], although genome-wide analyses have implicated complex deregulatory events such as enhancer hijacking, leading to Telomerase reverse transcriptase (*TERT*) over-expression and *ATRX* inactivation in non-MNA high-risk neuroblastoma [3], [4]. However, there still remain non-MNA high-risk neuroblastomas for which oncogenic drivers remain unclear, even taking into account activating point mutations of the Anaplastic Lymphoma Kinase (*ALK*) gene, which are apparent in less than 10% of neuroblastomas, encompassing the non-MNA and MNA high risk subtypes [5]. As a consequence of the lack of common druggable targets, chemotherapy for neuroblastoma still relies largely on DNA damaging agents and inhibitors of mitosis [6]. We therefore sought to rationalize a broader, but relatively specific target class for new neuroblastoma therapeutics.

Mutant ALK has been shown to signal through the Mitogen-activated protein kinase (MAPK) pathway [7], and although only a low level of RAS-MAPK pathway mutations were reported in neuroblastoma diagnosis samples [8], recent sequencing analyses have shown that RAS pathway mutations are increased in relapsing neuroblastoma [9], [10]. Together *ALK* and *RAS* mutations implicate mitogen/extracellular signal-regulated kinases (MEK1/2) and extracellular signal-regulated kinases (ERK1/2) in survival and proliferation of neuroblastoma. Additionally, we recently demonstrated an unexpected role for the leucine G-protein coupled receptor (LGR5) as a critical upstream regulator of MEK-ERK signaling and cell survival of different neuroblastoma genetic subtypes, including *ALK* and *NRAS* mutant lines. Depletion of LGR5 in these lines led to dramatic attenuation of phosphorylation of MEK1/2 and ERK1/2 and an increase of BimEL, an apoptosis facilitator downstream of ERK, leading to apoptosis [11]. Based on the accumulating evidence for MAPK pathway involvement in neuroblastoma, we hypothesized that transcriptional mediators of the Ras-MEK-ERK pathway, specifically ETS-related transcription factors [12], [13] may represent a new target class for high-risk neuroblastoma. These transcription factors, including ETV1, can activate a RAS/ERK-regulated gene expression program in the absence of ERK activation [14] and have also been shown to be downstream of ALK signaling [7], [15]. Here we report evaluation of two ETS-family inhibitors, BRD32048, an inhibitor of ETV1 [16], and YK-4-279, an inhibitor of EWS-FLI, ERG and ETV1 [17], [18]. We demonstrate that YK-4-279 triggers apoptosis in a wide variety of neuroblastoma cell lines at low micromolar concentrations, but does not affect normal cells. Surprisingly, however, YK-4-279 does not directly affect MEK/ERK signaling, as might be expected from the ETS-Ras/MAPK association, but rather disrupts mitosis. Importantly, we further demonstrate that YK-4-279 can overcome multidrug resistance, and also synergize with mitotic inhibitors such as vincristine and MLN8237, an inhibitor of Aurora kinase A.

## Materials and methods

### Anticancer compounds and inhibitors

YK-4-279, vincristine, paclitaxel, doxorubicin, etoposide, topotecan, temozolomide, busulfan, cyclophosphamide, trametinib and alisertib (all from Selleckchem), melphalan (Insight Biotechnology) and cisplatin (Santa Cruz Biotechnology) were prepared in DMSO and stored at −20 °C. Epidermal growth factor and QVD (quinolyl-valyl-*O*-methylaspartyl-(-2,6-difluorophenoxy)- methyl ketone) were purchased from Sigma.

### Cell lines

Neuroblastoma cell lines used for this study were acquired from several resources; Kelly, LAN-1, SK-N-BE(2)-C and IMR32 (Pramila Ramani, Bristol Royal Infirmary), SK-N-AS, GIMEN, SH-SY5Y, NF-TERT and NF-730 (Carmel McConville, fibroblasts from Grant Stewart, University of Birmingham), CHP-212, SHIN, SHEP (Robert Ross, Fordham University), and RPE-1 h-TERT (ATCC). For resistant lines, Kelly were obtained from ECACC (Salisbury, UK) and SK-N-AS were from ATCC (Manassas, VA, USA). The vincristine-resistant sub-lines SK-N-AS^r^VCR^20^ [19] and Kelly^r^VCR^10^ were established by continuous exposure to increasing drug concentrations as previously described [20] and obtained from the Resistant Cancer Cell Line (RCCL) collection (www.kent.ac.uk/stms/cmp/RCCL/RCCLabout.html). All neuroblastoma cell lines were mycoplasma-free and authenticated using the Promega PowerPlex 21 system (Eurofins, Germany). Most of the cell lines were cultured in Dulbecco's modified Eagle's medium (DMEM) mixture F12-HAM (Sigma) supplemented with 10% fetal bovine serum (FBS) (Life Technologies), 200 mM l-Glutamine (Sigma), 100 units/ml penicillin, 0.1 mg/mL streptomycin (Sigma) and 1% (v/v) non-essential amino acids (Life technologies). GIMEN, LAN1, Kelly, NF-730 and NF-TERT were cultured in RPMI (Life Technologies).

For imaging studies, lines were transduced with lentivirus constructed by excising the histone H2B gene from the PGK-H2B-mCherry vector (Addgene), and inserting it into the PGK-GFP vector (Addgene) to produce GFP-H2B lentivirus. Lentiviral stocks were produced in HEK 293 cells, using standard protocols. Transductions were selected for GFP-H2B expressing cells by flow cytometry.

### Proliferation and colony formation assays

For MTT assays, 3000–15000 cells were seeded in 80 μl of media. Next day 20 μl (5 times concentrated) of several drug concentrations prepared in 1:2 serial dilutions were added in triplicates. After 72 h, 10 μl of MTT (5 mg/ml) (Sigma) was added and incubated for 3 h. Thereafter, 50 μl of SDS lysis buffer (10% SDS ^w^/_v_, 0.01 M HCl, pH 5.5) was added and incubated overnight. MTT absorbance was read using SpectraMax 190 plate-reader (Molecular Devices) at 570 nm. 650 nm was used as a reference wavelength. Percentage survival for each dose was calculated by multiplying absorbance values by 100 and dividing by control absorbance value. Dose-response curves were drawn using GraphPad Prism which were used to determine IC_50_ values.

For clonogenic assays, 1000 cells were seeded in 6 well plates. After two days compounds were added. Media and compounds were replenished every four days. The plates were incubated until several colonies appeared. For colony staining, media was aspirated followed by PBS wash. Colonies were fixed by buffered neutral 10% formalin (Sigma) for 2 h and washed with PBS three times. Methylene blue (0.3% (w/v), Sigma) was added to wells and incubated overnight. Plates were then washed several times with water to remove excessive stain and photographed.

### Flow cytometry/cell cycle analysis

After drug treatments, medium was collected in 15 ml tubes. Cells were trypsinized and pooled in 15 ml tubes. Cells were centrifuged at 2000 rpm for 5 min at 4 °C followed by media removal. Cells were washed in PBS once, fixed in 70% ice cold ethanol and stored at −20 °C until analysis. Cells were washed once with PBS, aspirated and resuspended in 100 μl of RNase A solution (Qiagen 1:1000) and incubated at 37 °C for 15 min. Finally 400 μl of propidium iodide (Sigma: 50 μg/ml) was added, transferred to flow tubes and incubated at 37 °C for 15 min in the dark. Cells were analyzed using a BD LSRFortessa. Cell cycle data analysis was carried out using FlowJo software v10.

For combinatorial cell-death and mitosis analysis using Zombie/phospho-histone H3 (ser10) staining, 2 × 10^6^ cells were seeded in 10 cm dishes. After double thymidine synchronization, YK-4-279 was added and further incubated for 24 h. The medium was collected and cells detached with Accutase (Millipore). Cells were pooled with medium and centrifuged (1500 rpm for 5 min). After washing with PBS, cells were stained with Zombie NIR live-dead stain (BioLegend, 1:2500) for 15 min in the dark. Subsequently cells were fixed (0.5% ^v^/_v_ paraformaldehyde, 15 min) and permeabilized (0.1% Triton X-100 ^v^/_v_, 15 min). Cells were stained with phospho-histone H3 (ser10) (1:1000) antibody for 45 min in dark followed by secondary Alexa 488 antibody for 30 min (Invitrogen). Antibodies were prepared in PBS containing 1% FBS. Finally cells were suspended in Propidium iodide/RNase A as described in cell cycle analysis. Stained samples were analysed using a BD LSRFortessa.

### Immunoblots, Immunofluorescence and antibodies

Whole cell lysates were prepared using RIPA lysis buffer (25 mM Tris-HCl pH 8.0, 150 mM NaCl, 1 mM EDTA, 0.2% SDS, 0.5% NP40 and 0.2% sodium deoxycholate) supplemented with protease and phosphatase inhibitors (Roche). Cells while in media were collected in 15 ml tubes, centrifuged at 2000 rpm for 5 min at 4 °C, media aspirated and cells washed with PBS. Lysis buffer was added and samples were sonicated (Diagenode bioruptor) and clarified at maximum speed at 4 °C for 20 min. Protein quantification was performed using the Micro BCA kit (Thermo Fischer Scientific) and proteins were resolved by SDS-PAGE and transferred to PVDF membranes (Millipore). Blots were developed using KPL LumiGLO Peroxidase Chemiluminescent substrate. Antibodies used were phospho-Erk1/2 (4370S, 1:1000), Erk1/2 (9102s, 1:1000), phospho-histone H3 (ser10) (9701, 1:2000), Cyclin B1 (4138, 1:1000), phosho-Aurora A (T288) (3079, 1:500), Aurora A (3092, 1:500), Aurora B (3094, 1:500), Acetyl-α-tubulin (Lys40) (5335, 1:1000), α-tubulin (2144S, 1:10000), cleaved caspase-3 (9664, 1; 1000) (all from Cell Signaling Technology), cleaved PARP (ab32064, 1:3000), Histone H3 (ab10799, 1:3000) (all from Abcam), p53 (SC-126, 1:1000), p21 (OP64, Oncogene, 1:500) and beta-actin-peroxidase (A3854, Sigma, 1:40000). Peroxidase conjugated secondary antibodies were purchased from Sigma.

To assess YK-4-279 effects on Ras-MEK/ERK signaling, cells were seeded in media containing 1% FBS and incubated for 24 h. Cells were subsequently treated with DMSO or YK-4-279 (SKNAS: 1 μM, GIMEN: 2 μM) or Trametinib (50 nM) for 4 h. Finally cells were treated with PBS or EGF (200 ng/ml) for further 10 min and whole cell lysates were prepared for immunoblotting.

For immunofluorescence, 15,000 cells were seeded in 8-well glass chambers and synchronized by double thymidine block. After release from second thymidine block, cells were treated with DMSO or 2 μM of YK-4-279 for 7 h. Media was aspirated and washed with PBS twice before paraformaldehyde (2% v/v in PBS) fixing for 10 min. After washing with PBS, cells were permeabilised using 0.1% Triton X-100 (v/v) for 10 min. After further washing with PBS, cells were blocked with 1% BSA (w/v) in PBS for 30 min, and then incubated with antibodies at room temperature for 2 h. Antibodies used were Aurora A (Cell signaling Technologies, 3092, 1:250), PLK1 (Bethyl Laboratories, A300-252A, 1:500), Eg5 (BioLegend, 627801, 1:250) and α-tubulin (Thermo Scientific, 62204, 1:100) After three washes with PBS, labelled secondary antibodies (Invitrogen 1:1000) were added and incubated in the dark at room temperature for 45 min. Cells were washed with PBS, DAPI added to cells, and incubated in the dark for a further 10 min. DAPI was then removed, cells were washed with PBS, and images acquired using spinning disk confocal microscope CV1000 (Olympus Life Science).

### RNA interference

Transient knockdown was performed using Lipofectamine RNAiMAX (Invitrogen). 0.3 × 10^6^ cells were seeded in 60 mm dishes after reverse transfection carried out using 50 nM siRNA for 72 h. Opti-MEM media (Invitrogen) was used for diluting siRNA and RNAiMAX. After 48 h of incubation with siRNA, YK-4-279 was added and cell lysates were harvested after 24 h for immunoblotting. The siRNA sequence used for p53 was 5′-CAGCAUCUUAUCCGAGUGGAA-3’. A non-targeting siRNA, 5′-UGGUUUACAUGUUUUCUGA-3′ was used as a negative control.

### Live cell imaging

For real-time proliferation assays, SK-N-AS (5000 cells/well), SK-N-BE(2)-C (5000 cells/well), Kelly (10,000 cells/well), NF-TERT and NF-730 (3000 cells/well), and RPE-1 (3000 cells/well) were seeded in 96 well plates in triplicates. After overnight incubation, different concentrations of YK-4-279 or BRD32048 were added and immediately incubated in IncuCyte ZOOM live cell imaging system. Phase confluence was measured every two hours. IncuCyte system scanned and took images at four different fields in each well. At the end of the assay raw values were exported into GraphPad Prism and plotted as percentage phase confluence (representative of growth) against time.

For visualization of mitosis, 100,000 cells previously transduced with GFP-H2B lentivirus were seeded in 35 mm glass bottom Petri dishes. After 24 h incubations cells were synchronized using the double thymidine block method. After the second thymidine block, cells were washed with PBS twice and refreshed with medium without thymidine and immediately added DMSO or YK-4-279 and incubated for 6 h. Images were acquired at 60× magnification on a widefield light microscope (DeltaVision), at 37 °C and 5% CO_2_. GFP was excited using a wavelength of 488 nm, and tubulin was visualized using SiR-Tubulin (Spirochrome, CY-SC002), at an excitation wavelength of 647 nm, where cells were stained with 1 μM SiR-Tubulin for 1 h before imaging. Prometaphase cells were identified based on having compact chromatin, and lacking a diffuse nuclear GFP signal after the nuclear envelope had broken down, but without alignment into metaphase. Other phases were identified as follows: Metaphase - chromosomes aligned, anaphase - chromosomes segregated, telophase - chromosomes segregated with signs of nuclear envelope reassembly and loss of classic mitotic chromosome appearance. Cells (n > 25) in a minimum of 15 fields of view were scored in triplicate treatments by two independent assessors. In the case of videos, frames were taken every three minutes for a period of one hour.

### Drug combination studies

Drug interactions were analyzed by the method developed by Chou and Talalay. IC_50_ values for all compounds used for combination studies were determined prior to experiment setup. Equipotent ratios of two compounds were prepared across a wide range of concentrations (sub-lethal to lethal) and the cells treated for 72 h. Percentage survival values were converted into Fa (fraction affected) values using the formula, 1-(% survival/100). These values were fed into CompuSyn software to determine combination index values (<1: synergy, = 1: additive, >1: antagonistic).

## Results

### Differential efficacy of the ETV1 inhibitors BRD32048 and YK-4-279 on neuroblastoma cell growth

**Fig. 1:**
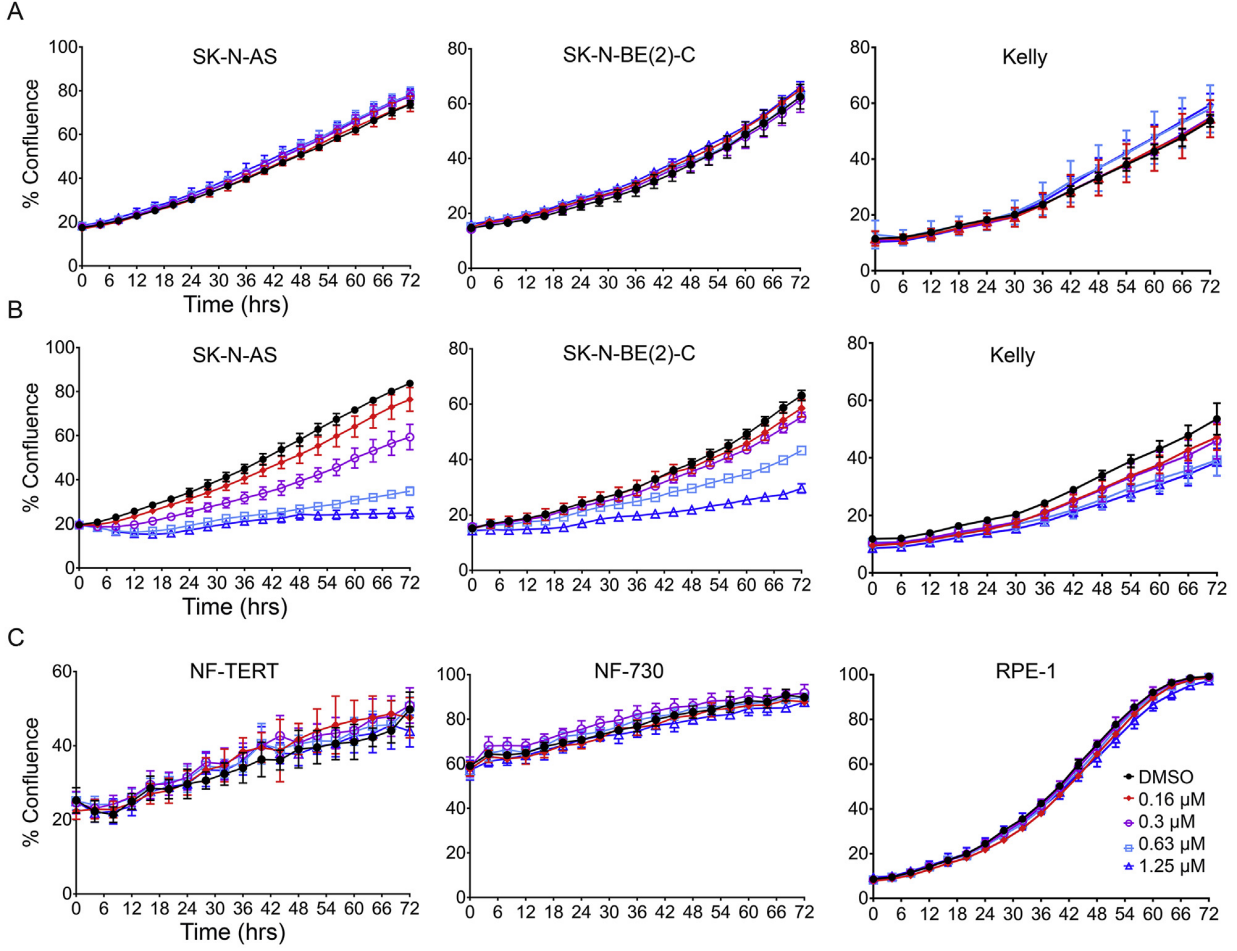
YK-4-279 suppresses neuroblastoma cell growth. Real-time cell growth analysis following BRD32048 treatment using Incucyte Zoom live cell imaging. Phase confluence of cells is plotted over time. **(A)** BRD32048 treatment of neuroblastoma lines, **(B)** YK-4-279 treatment of neuroblastoma lines, **(C)** Normal human non-cancer cell lines treated with YK-4-279. Live cell imaging was carried out in 96-well plates in three technical replicates (n = 3; mean ± SD).

**Fig. S1:**
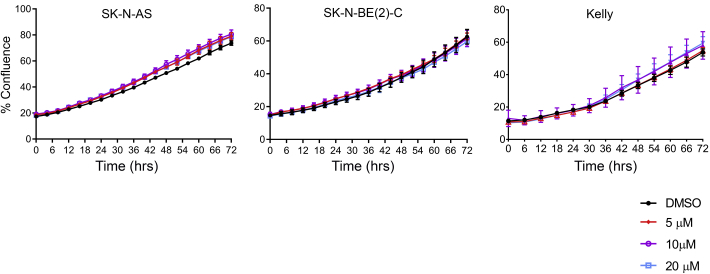
BRD32048 activity on neuroblastoma cell lines. Neuroblastoma cell lines were seeded in 96-well plates and after overnight incubation, cells were treated with 2-fold serial dilutions of BRD32048. Real-time cell growth analysis after BRD32048 treatment using Incucyte Zoom live cell imaging system. Growth of neuroblastoma cell lines was not affected even at highest concentrations tested.

BRD32048 was reported to act as a perturbagen of ETV1 [16] while YK-4-279 was initially characterized as a disruptor of the EWS-FLI/DHX9 interaction [17], and later shown to also affect ERG and ETV1 [18], [21]. Our initial screening of these compounds showed that BRD32048 treatment of SK-N-AS, SK-N-BE(2)-C and Kelly neuroblastoma cell lines had no effect on cell proliferation (Fig. 1A), even at high micromolar concentrations (Supplementary Fig. S1). In contrast, YK-4-249 reduced cell growth in a dose-dependent manner at sub-to low-micromolar concentrations (Fig. 1B). YK-4-279 did not inhibit proliferation of normal human fibroblasts and epithelial cell lines namely, NF-TERT, NF-730 and RPE-1 (Fig. 1C). The discrepant effects of these two ETV1 inhibitors suggests that the primary mode of action of YK-4-279 may, contrary to the literature and our expectations, be independent of the Ras-MEK/ERK-ETS(ETV1) axis.

### YK-4-279 inhibits neuroblastoma cell growth independent of MEK-ERK signaling

**Fig. 2:**
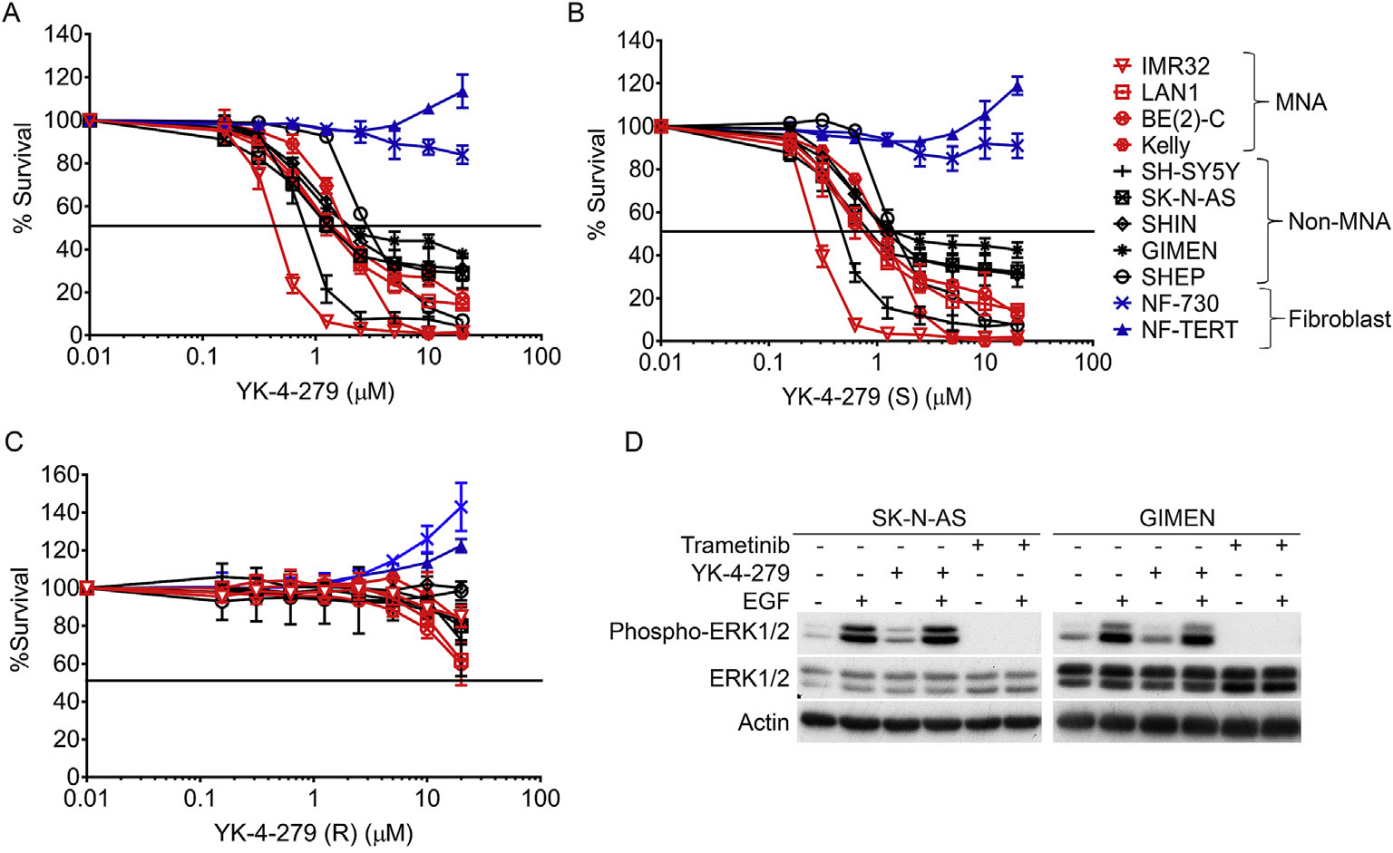
Enantiomer-specific YK-4-279 inhibition of neuroblastoma cell lines. **(A)** Nine neuroblastoma cell lines and two non-cancerous cell lines were further screened by MTT based cell proliferation assay to determine YK-4-279 sensitivity and IC_50_ values. **(B)** Dose-response curves of YK-4-279(S) enantiomer on neuroblastoma cell lines. **(C)** Activity of the YK-4-279(R) enantiomer on neuroblastoma cell lines. All MTT assays were carried out in three independent biological replicates each having three technical replicates (n = 3; mean ± SD). **(D)** Immunoblotting of SK-N-AS and GIMEN cells to assess effects on EGF-induced MEK/ERK signaling. Cells were treated with YK-4-279 or Trametinib for 4 h, and then PBS or EGF for a further 10 min before assessing ERK phosphorylation.

We next established the IC_50_'s for YK-4-279 in a panel of nine neuroblastoma cell lines. All these lines were sensitive to YK-4-279 (Fig. 2A), with IC_50_'s ranging from 0.4 to 2 μM. This range is similar to that reported for Ewing's sarcoma cell lines (0.5–2 μM) [17]. In order to further explore the selectivity of YK-4-279 action, we tested YK-4-279(R) and YK-4-279(S) enantiomers [22]. Neuroblastoma cell line sensitivity to YK-4-279(S) was approximately two-fold greater compared to the racemic mix, while neuroblastoma cell lines were not sensitive to the (R)-enantiomer (IC_50_: >20 μM) (Fig. 2A–C and Table 1). With regard to the YK-4-279 sensitivity profile, no obvious segregation attributable to a particular oncogenic genotype, such as *MYCN* amplification or mutant *NRAS* (SK-N-AS) was apparent (Table 1). This further suggests that sensitivity to YK-4-279 is not restricted to the Ras-MEK/ERK-ETS axis. In order to directly evaluate this, we treated SK-N-AS and GIMEN lines with epidermal growth factor (EGF) to stimulate MEK/ERK signaling, and assessed whether YK-4-279 could inhibit the increase of phosphorylated ERK that accompanies activation of this pathway. Whilst YK-4-279 was not able to attenuate ERK phosphorylation, the MEK inhibitor Trametinib totally eliminated ERK phosphorylation after EGF treatment (Fig. 2D). Together with our data above, this experiment demonstrates that the primary mode of action of YK-4-279 is independent of the Ras-MEK/ERK-ETS axis.

**Table 1:** MTT assay based IC_50_ (μM) values were determined for nine neuroblastoma cell lines and two normal fibroblasts. Values are means ± SD of three independent experiments.

Cell Line	Known genotype	YK-4-279: IC_50_ ± SD	YK-4-279(S): IC_50_ ± SD
IMR32	MNA	0.44 ± 0.03	0.27 ± 0.005
SH-SY5Y	*ALK* mutation	0.81 ± 0.06	0.48 ± 0.03
SK-N-AS	*NRAS* mutation, *NF-1* deletion and *TP53* truncation	1.30 ± 0.21	0.84 ± 0.08
LAN1	MNA, *ALK* mutation, *TP53* truncation	1.35 ± 0.23	0.78 ± 0.02
SK-N-BE(2)-C	MNA, *TP53* mutation	1.42 ± 0.11	0.81 ± 0.26
Kelly	MNA, *ALK* mutation, *TP53* mutation	1.76 ± 0.09	1.14 ± 0.1
SHIN	–	1.98 ± 0.28	1.33 ± 0.32
GIMEN	*NF-1* deletion	2.00 ± 0.00	1.64 ± 0.26
SHEP		2.93 ± 0.05	1.42 ± 0.05
NF-730	–	>20	>20
NF-TERT	–	>20	>20

MNA: MYCN amplification, IC_50_ (μM): Half maximal inhibitory concentration.

### YK-4-279 induces mitotic arrest and apoptosis in neuroblastoma cells

**Fig. 3:**
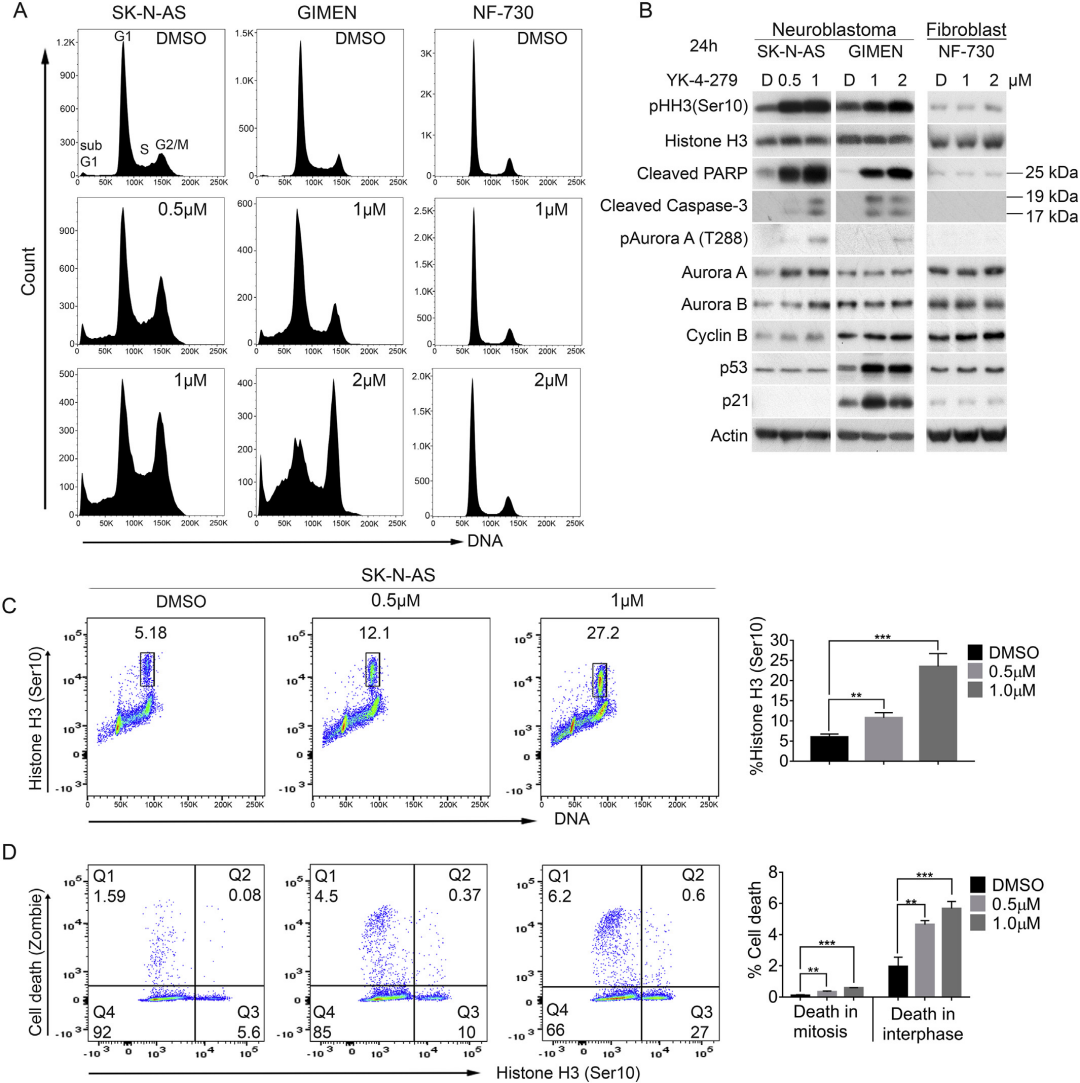
YK-4-279 induces mitotic arrest and apoptosis in neuroblastoma cell lines. **(A)** Propidium-iodide based cell cycle analysis of YK-4-279 treated cells using flow cytometry. Cells were treated for 24 h with 0.5× and 1xIC_50_ concentrations. Cell cycle analysis was carried out in three independent biological replicates. **(B)** SK-N-AS, GIMEN and NF-730 were treated with DMSO (D) or two doses of YK-4-279 for 24 h and protein lysates harvested. Blots were probed for several cell cycle and cell death markers. All neuroblastoma cell lines showed increase of phospho-histone H3 (Ser10(pHH3), cleaved PARP and active caspase-3. Actin was used as a loading control. **(C)** Flow cytometry with triple staining for phospho-histone H3 (Ser10) (mitotic cells). Dot plot showing DNA content (X-axis) against phospho-histone H3 (Y-axis). Quantification bar graphs of percentage mitotic cells (right side). Data shown as mean ± SD of three technical replicates. **(D)** Zombie live/dead cell marker versus phospho-histone H3(Ser10). Cells synchronized using double-thymidine block were treated with YK-4-279 for 24 h after release. Cells were stained with Zombie dye followed by phospho-histone H3 (Ser10) after fixation and permeabilization and subsequently with propidium iodide. Dot plots of phospho-histone H3 positive cells against dead cells. Q1 and Q2 represent percentage dead cells (Q1 and Q2) negative or positive for phospho-histone H3, respectively. Data shown as mean ± SD of three technical replicates. **P < 0.01, ***P < 0.001. Unpaired Student t-test was used to assess the significance of difference between the control and treated samples.

**Fig. S2:**
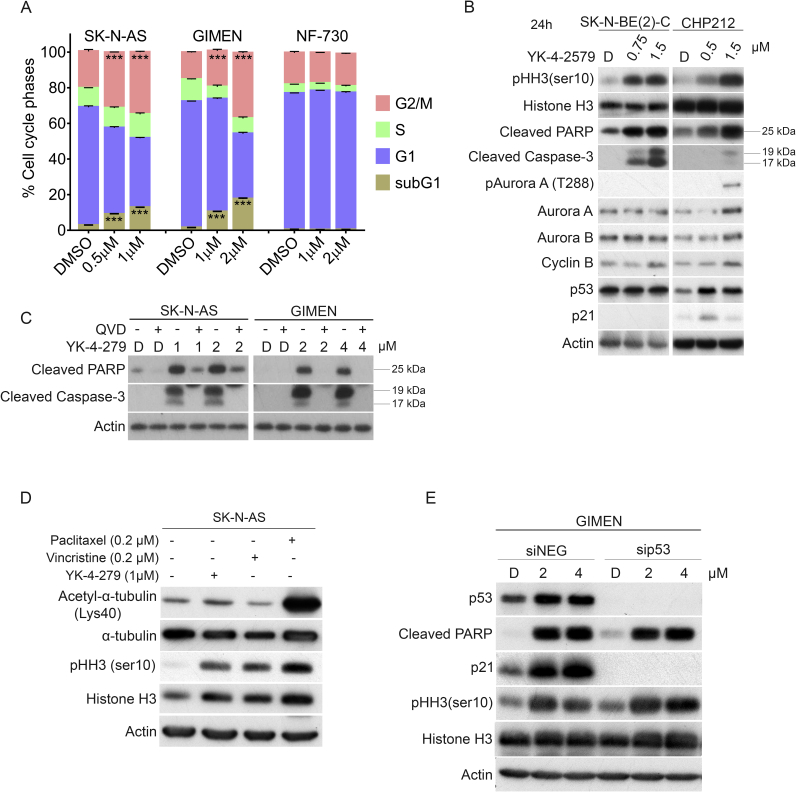
YK-4-279 induces mitotic arrest and apoptosis. **(A)** Quantification of percentage of cells at various phases of cell cycle after treatment with DMSO or YK-4-279 for 24 h. Error bars represent ±SD of three independent replicates. **P < 0.01, ***P < 0.001. Unpaired Student t-test was used to assess the significance of difference between the control and treated samples. **(B)** SK-N-BE(2)-C and CHP-212 were treated with DMSO (D) or two doses of YK-4-279 for 24 h and cell lysates were harvested in RIPA buffer. Blots were probed for several cell cycle and cell death markers. All neuroblastoma cell lines showed increase of Histone H3 (Ser10), cleaved PARP and active caspase-3. Actin was used as a loading control. **(C)** Immunoblots demonstrating that YK-4-279-induced cell death of SK-N-AS and GIMEN cells is rescued by QVD treatment. Note decreased cleaved caspase-3 and cleaved PARP after QVD treatment. **(D)** Immunoblotting for acetyl-α-tubulin (Lys40) (indicator of tubulin microtubule stabilization) and phospho-histone H3(ser10) (pHH3). α-tubulin and actin were used as loading controls. **(E)** Knockdown of wildtype p53 in GIMEN and assessment of YK-4-279 activity. Cells were reverse transfected with either control siRNA or p53 siRNA. After 72 h of incubation whole cell lysates were harvested for western analysis and probed for mitotic and apoptotic markers.

In order to gain mechanistic insights into the profound effects of YK-4-279 on neuroblastoma cell proliferation and survival, we conducted cell cycle analyses. After treatment of SK-N-AS and GIMEN lines at 0.5× and 1× IC_50_ values for 24 h, a strong dose - dependent G2/M arrest was apparent in both neuroblastoma cell-lines, but not in normal human fibroblasts (NF-730). We also observed a marked increase in sub-G1 population, indicative of cell death (Fig. 3A & Fig. S2A). Immunoblotting of corresponding cell lysates confirmed that YK-4-279 treatment leads to increased PARP cleavage, detected by the 25 kDa caspase-3 dependent fragment, thereby confirming apoptosis, as well as accumulation of phospho – histone H3 (serine 10) (pHH3), a marker of chromatin condensation and early mitosis (Fig. 3B). YK-4-279 also induced mitotic arrest and apoptosis in SK-N-BE(2)-C and CHP-212 cells (Fig. S2B). Caspase-3 dependent apoptosis of SK-N-AS and GIMEN lines was further confirmed by the detection of 17 and 19 kDa cleaved caspase-3 fragments and inhibition of apoptosis by QVD treatment (Fig. S2C). The dramatic induction of pHH3, cleaved PARP and cleaved caspase-3 was not apparent in NF-730 normal fibroblast cells (Fig. 3B).

As inhibition of Aurora kinases and polo-like kinases can result in a similar mitotic phenotype as observed with YK-4-279, their levels were also assessed by immunoblotting. Neither Aurora A or B levels were diminished after YK-4-279 treatment, and nor was their activity, as histone H3 phosphorylation, which depends on Aurora B [23], is not inhibited. Aurora A kinase activity is also not diminished, as we observed an increase in auto-phosphorylated Aurora A (T288) [24] (Fig. 3B). This data clearly indicates that kinase activity of Aurora kinases is not the direct target for YK-4-279, but rather that they can be considered as markers of the effects of YK-4-279 on the cell cycle. We also assessed the levels of cyclin B, which is proteosomally degraded during metaphase [25]. Cyclin B levels were not affected by YK-4-279 treatment, which, together with the mitotic kinase evaluation above, suggests that cells were retained in early mitosis and were not able to proceed into metaphase.

To gain further mechanistic insight into the mode of action of YK-4-279, we compared its effects with the classical mitotic inhibitors paclitaxel and vincristine, which bind tubulin and/or microtubules, leading to over-stabilization and destabilization respectively, and ultimately inhibition of mitosis [26]. Paclitaxel promotes hyper-polymerization of α-tubulin which can be monitored by increased acetyl-α-tubulin (Lys40) (a marker of tubulin stabilization) [27]; conversely, de-acetylation is observed in response to vincristine, which inhibits tubulin polymerization [28]. YK-4-279 did not increase or decrease acetylation of microtubules significantly, thereby ruling out tubulin binding as a mechanism of action for YK-4-279 (Fig. S2D), and suggesting that YK-4-279 is targeting a different regulatory step to spindle poisons.

We next investigated whether the apoptosis induced by YK-4-279 occurs in mitosis or in interphase. After synchronizing cells by double thymidine block, we treated with YK-4-279 for 24 h and labelled cells with propidium iodide, pHH3, and Zombie (a live/dead cell marker). Consistent with our immunoblot analyses, our flow-cytometry data showed that YK-4-279 induced accumulation of pHH3 in a dose-dependent manner (Fig. 3C). By plotting pHH3 versus cell death after YK-4-279 treatment, we observed that a small but significant percentage of pHH3 - positive cells stained positive for the cell death marker (Q2) (Fig. 3D). However, most of the dead cells are negative for pHH3 (Q1), indicating that only a small fraction of cells died in mitosis, whereas the majority of cells died in subsequent interphase. We also found that YK-4-279 could activate apoptotic checkpoints in a p53-independent manner. Although YK-4-279 induces high levels of wild-type p53 in GIMEN cells, together with elevated p21 and cleaved PARP, it induces apoptosis in both p53 proficient (GIMEN, CHP-212) and deficient cell lines (SK-N-AS, SK-N-BE(2)-C) (Fig. 3B, Fig. S2B & Table 1). To confirm this, we knocked down wild-type p53 in GIMEN cells, and despite the absence of p53, we could still see induction of apoptosis, with equivalent levels of cell death in both control and p53 knockdown cells (Fig. S2E). Taken together, our data demonstrate that YK-4-279 leads to mitotic arrest and can induce p53-independent apoptosis.

### YK-4-279 disrupts early mitotic regulatory events

**Fig. 4:**
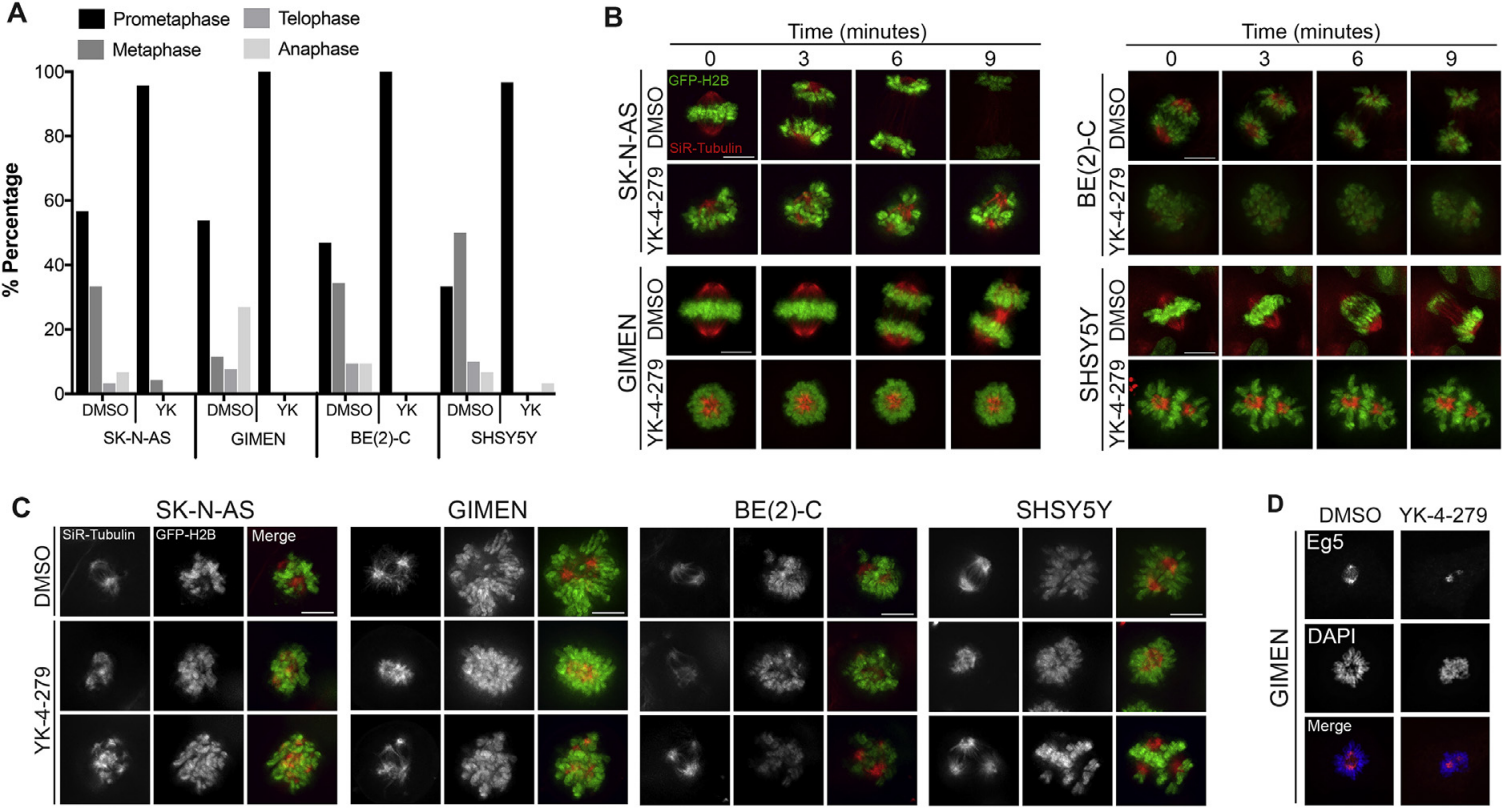
Disruption of mitosis induced by YK-4-279. **(A)** Four neuroblastoma cell lines, SK-N-AS, GIMEN, SK-N-BE(2)-C and SH-SY5Y were treated DMSO or with 1 μM YK-4-279 (5 μM was used for GIMEN). Prior to treatment cells were synchronized by standard double thymidine block protocol. Immediately after release, cells were treated with YK-4-279 for six hours. The graph shows the percentage of cells at distinct phases of mitosis, which were scored based on chromatin organization, in three independent replicates (n > 25). **(B)** SK-N-AS, GIMEN, SK-N-BE(2)-C and SH-SY5Y representative mitotic structures (Red: microtubules, Green: chromosomes) from three independent replicates. Diverse microtubule organization is evident from the images. All DMSO treated cells showed typical metaphase equatorial plates and progressed through mitosis and segregated chromosomes within the nine-minute time frame, whereas YK-4-279 cells were mostly stuck at prometaphase. **(C)** Static images of four live neuroblastoma cell lines. The range of abnormalities include fragmented (SK-N-AS), unseparated (GIMEN) and multipolar spindles (SK-N-BE(2)-C and SH-SY5Y) (Red: microtubules, Green: chromosomes). Images shown were representative of three independent replicates (n > 25). See also videos in Supplementary information. **(D)** Confocal microscopy showing immunofluorescent localization of Eg5 (pink) in YK-4-279-treated GIMEN cells. Chromatin is stained with DAPI (blue).

**Fig. S3:**
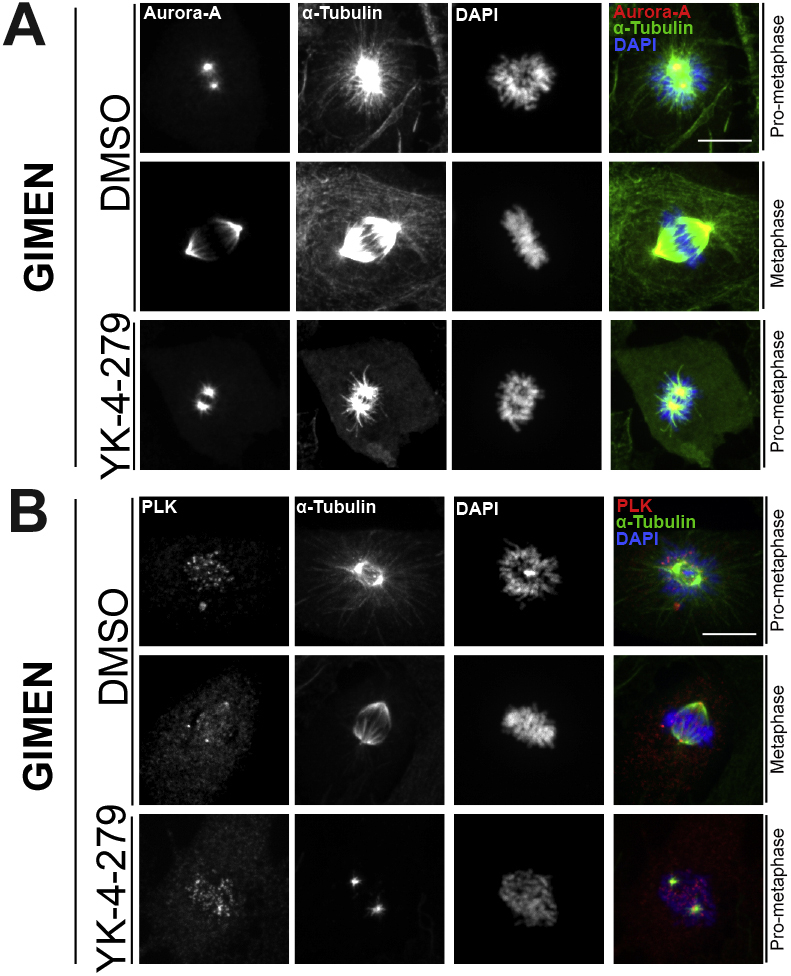
Localization of key mitotic kinases in early mitosis. GIMEN cells were treated with YK-4-279 and stained with Aurora A, PLK1 and α-tubulin antibodies. Chromatin was visualized using DAPI. **(A)** Aurora A localised to centrosomes and spindle tubules in both DMSO and YK-4-279 indicating no abnormalities in sub-nuclear distribution. **(B)** PLK1 staining showing a similar pattern. Tubulin staining revealed appearance of normal astral microtubules.

**Fig. S4:**
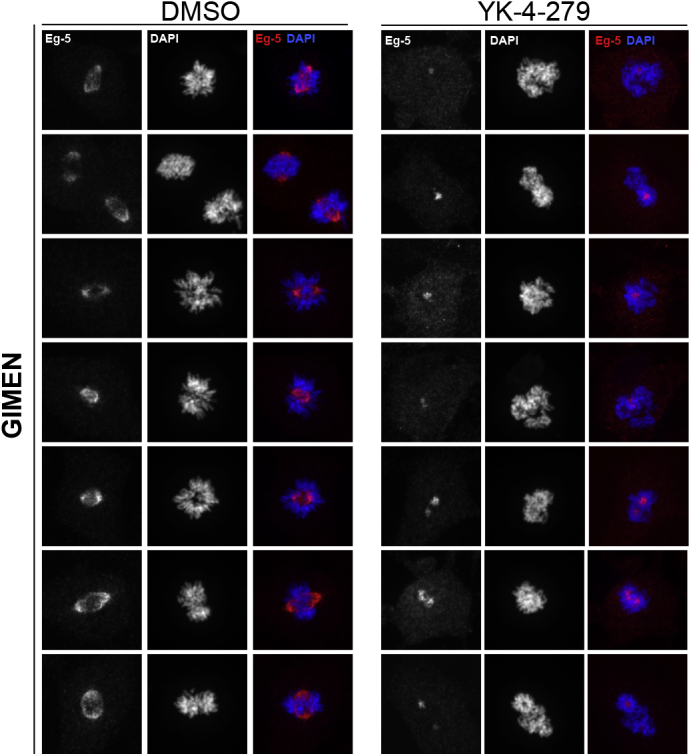
Effect of YK-4-279 on Eg-5 localization. Confocal microscopy of Eg-5 immunostaining shows reduced accumulation of Eg-5 on spindle. Multiple early mitotic structures are shown.

Our findings suggest that YK-4-279 treatments interfere with progression through mitosis. To further evaluate this, we performed live cell imaging to visualize chromatin (with GFP-histone H2B) and microtubules (with SiR-tubulin, a tubulin-binding fluorogenic probe) [29] in cells treated with YK-4-279 after release from G1/S-phase synchronization. We observed that while control cells were at different stages of mitosis, YK-4-279 treated cells were predominantly in pro-metaphase (Fig. 4A). To probe this further, we performed time-lapse imaging of single mitotic cells. This confirmed that while all control cells transitioned from pro-metaphase to telophase, YK-4-279-treated cells failed to progress through mitosis and remain largely arrested in prometaphase (Fig. 4B and Supplementary videos 1–8), consistent with our immunoblotting of cyclin B and pHH3 (Fig. 3B). This may be attributable to defects in spindle assembly and/or dynamics, which is supported by our analyses revealing that cells treated with YK-4-279 displayed abnormalities in their mitotic spindles, including unseparated spindle poles (centrosomes), fragmented spindles and multipolar spindles (Fig. 4C). To gain further insights into the mechanisms underlying YK-4-279-induced spindle defects, we examined the localization of key mitotic kinases. Both Aurora-A and Polo-like kinase (PLK) orchestrate progression through mitosis by regulating microtubule spindle assembly. GIMEN cells treated with YK-4-279 (thus arrested in pro-metaphase) had normal localization of both Aurora-A (Fig. S3A) and PLK (Fig. S3B) at centrosomes and kinetochores, respectively. Further, control cells progressed to metaphase, wherein Aurora-A spreads from centrosomes to mark polar and kinetochore microtubules (Fig. S3A) and PLK starts accumulating at the centrosome (Fig. S3B). These observed re-localizations of Aurora-A and PLK cannot be examined in YK-4-279 treated GIMEN cells, as they are blocked at pro-metaphase. Notably, however, YK-4-279 treatment had no discernible effects on the formation of astral microtubules in pro-metaphase (Fig. S3); thus, raising the possibility that YK-4-279 may hinder the formation of polar and/or kinetochore microtubules that are required for transition from pro-metaphase to metaphase. Given that Eg5 kinesin is a key regulator of this process, we hypothesized that YK-4-279 may interfere with its function, and found that in DMSO-treated pro-metaphase cells Eg5 accumulates on the spindle, while YK-4-279 treatment appears to interfere with Eg5 distribution (Fig. 4D and Fig. S4). Together, these findings provide new mechanistic insights into how YK-4-279 interferes with spindle assembly/dynamics and mitotic progression, eventually inducing neuroblastoma cell death.

Supplementary videos related to this article can be found at http://dx.doi.org/10.1016/j.canlet.2017.05.027

The following is the supplementary data related to this article:

Video 1 GIMEN: DMSO treated. Video 1

Video 2 GIMEN: YK-4-279 treated. Video 2

Video 3 SH-SY5Y: DMSO treated. Video 3

Video 4 SH-SY5Y: YK-4-279 treated. Video 4

Video 5 SK-N-BE(2)-C: DMSO treated. Video 5

Video 6 SK-N-BE(2)-C: YK-4-279 treated. Video 6

Video 7 SK-N-AS: DMSO treated. Video 7

Video 8 SK-N-AS: YK-4-279 treated. Video 8

### YK-4-279 overcomes vincristine resistance and multidrug resistance

**Fig. 5:**
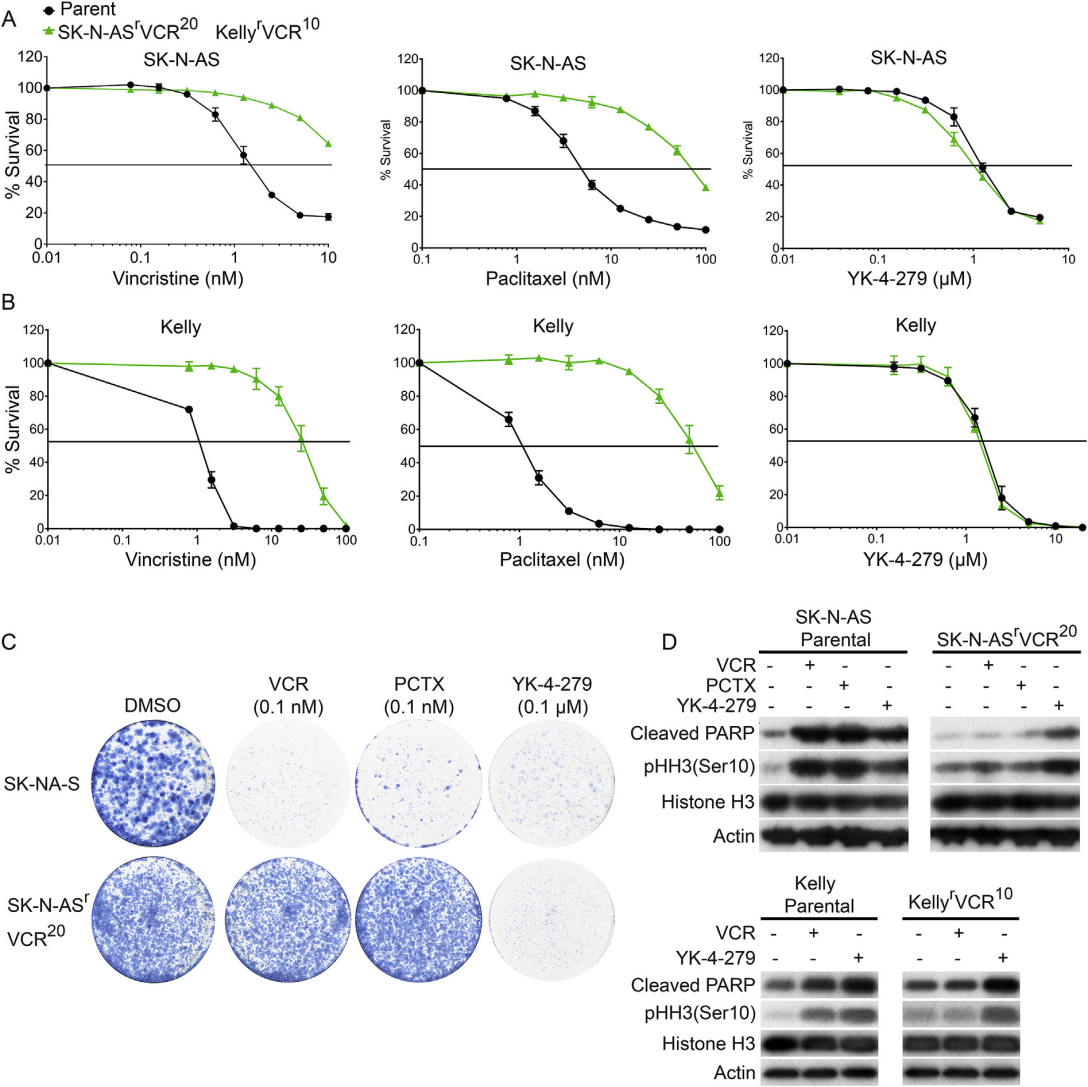
YK-4-279 circumvents resistance to vincristine. **(A)** Proliferation assays show that SK-N-AS^r^VCR^20^ are resistant to vincristine and paclitaxel, but not YK-4-279. **(B)** as above for Kelly ^r^VCR^10^ cells. Vincristine resistant cell lines and their parental counterparts assessed after 72 h of YK-4-279 treatment by MTT assay. Dose-response curves shown are representative of three independent replicates. **(C)** SK-N-AS and SK-N-AS^r^VCR^20^ lines were seeded in 6 well plates and treated with vincristine, paclitaxel and YK-4-279 with regular replenishments. After 18 days, cells were stained with methylene blue and imaged. **(D)** SK-N-AS^r^VCR^20^ and Kelly^r^VCR^10^ lysates were prepared 24 h after treatment with 2× IC_50_ concentrations of vincristine, paclitaxel and YK-4-279. Blots were probed for mitotic and apoptotic markers, demonstrating unimpaired efficacy of YK-4-279.

Vincristine is one of the most effective chemotherapeutic agents for neuroblastoma [30]. However neuroblastoma patients and cell lines acquire resistance to vincristine [31]. Given the probability that YK-4-279 acts through a mechanism other than microtubule polymerization, we assessed the potential of YK-4-279 in overcoming vincristine induced resistance in neuroblastoma cell line models. We tested YK-4-279 on SK-N-AS and Kelly cell lines with acquired vincristine – resistance, named SK-N-AS^r^VCR^20^ and Kelly^r^VCR^10^ respectively. Vincristine treatment did not result in typical sigmoidal-dose response even at the highest concentration tested on SK-N-AS^r^VCR^20^ cells (>7-fold resistant relative to the parental line) (Fig. 5A). Similarly, Kelly^r^VCR^10^ is 30-fold resistant to vincristine (Fig. 5B). These cell lines are also more resistant to paclitaxel (SK-N-AS = 14-fold; Kelly = 50-fold) than vincristine in terms of IC_50_ values. Strikingly both VCR resistant cell lines are as sensitive as their parental counterparts to YK-4-279 (Fig. 5A and B). We further tested several drugs (topotecan, doxorubicin, etoposide, cisplatin, melphalan, busulphan and temozolomide) that are routinely used as front-line therapy in neuroblastoma to evaluate multidrug-resistance phenotype. SK-N-AS^r^VCR^20^ is cross-resistant to almost all drugs we tested (6–19 fold), except cisplatin (1.5 fold) and temozolomide (1.04-fold) (Table 2). Similarly, Kelly^r^VCR^10^ is also cross-resistant to doxorubicin (3-fold), etoposide (3-fold), busulfan (1.7-fold), but not to temozolomide (1.2-fold) and almost equally sensitive to cisplatin, melphalan and topotecan as their parental counterparts. We did not see any inhibition of proliferation with Cyclophosphamide even at millimolar concentrations. This analysis demonstrates that only YK-4-279 and temozolomide can overcome VCR resistance in both SK-N-AS^r^VCR^20^ and Kelly^r^VCR^10^ cell lines, unlike many other currently used chemotherapeutic drugs.

**Table 2:** Resistance and cross-resistance profiles of SK-N-AS^r^VCR^20^ and Kelly^r^VCR^10^ resistant cell lines. IC_50_ values are means ± SD of three independent experiments. Values in parentheses represent resistance factor values indicative of fold-resistance. IC_50_ (μM) values of parent versus resistant cell lines were used to determine resistance factor values.

Compound	SK-N-AS	SK-N-AS^r^VCR^20^
YK-4-279	1.3000 ± 0.0900	1.080 ± 0.100 (0.8)
Vincristine	0.0015 ± 0.2300	>0.01 ± 0.000 (>7)
Paclitaxel	0.0050 ± 0.2900	0.072 ± 0.007 (14)
Topotecan	0.0053 ± 0.0002	0.100 ± 0.000 (19)
Doxorubicin	0.0600 ± 0.0005	>1.00 ± 0.000 (>17)
Melphalan	2.3600 ± 0.0700	43.20 ± 1.272 (18)
Etoposide	0.5300 ± 0.0800	3.750 ± 0.750 (7)
Busulfan	122.00 ± 4.9497	768.0 ± 55.86 (6)
Cisplatin	174.00 ± 11.718	261.0 ± 31.81 (1.5)
Temozolomide	486.50 ± 70.000	510.0 ± 51.61 (1.04)

Compound	Kelly	Kelly^r^VCR^10^
YK-4-279	1.5900 ± 0.1600	1.470 ± 0.060 (0.9)
Vincristine	0.0010 ± 0.0000	0.030 ± 0.003 (30)
Paclitaxel	0.0010 ± 0.0000	0.050 ± 0.007 (50)
Doxorubicin	0.0365 ± 0.0007	0.126 ± 0.012 (3.45)
Etoposide	0.3800 ± 0.0332	1.190 ± 0.084 (3.13)
Busulfan	85.000 ± 7.4246	146.0 ± 39.59 (1.72)
Temozolomide	195.00 ± 14.142	231.5 ± 13.43 (1.2)
Cisplatin	81.000 ± 10.465	93.00 ± 15.90 (1.15)
Melphalan	1.7200 ± 0.0282	1.910 ± 0.028 (1.11)
Topotecan	0.0062 ± 0.0000	0.006 ± 0.000 (0.96)

We also performed clonogenic assay to assess the ability of YK-4-279 to overcome resistance. In line with cell proliferation data, YK-4-279 (even at very low concentration) reduced the colony formation in both sensitive and resistant SK-N-AS cell lines equivalently, whereas SK-N-AS^r^VCR^20^ is highly resistant and cross-resistant to vincristine and paclitaxel respectively (Fig. 5C). In order to confirm that these effects of YK-4-279 were attributable to disruption of mitosis as observed in our cell-line panel, we treated parental and resistant cell lines with vincristine or paclitaxel or YK-4-279 at 2 × IC_50_ for immunoblotting. As expected vincristine, paclitaxel and YK-4-279 induced phosphorylation of Histone H3 and cleaved PARP in parental cell lines (Fig. 5D). However resistant counterparts did not undergo either mitotic arrest or apoptosis when treated with vincristine or paclitaxel. In contrast YK-4-279 induced mitotic arrest and apoptosis in resistant cell lines (Fig. 5D). This further supports that YK-4-279 has a novel mechanism for disrupting mitosis distinct to vincristine or paclitaxel. Together, our data shows that YK-4-279 overcomes vincristine resistance and its activity is not restricted by multidrug-resistance.

### YK-4-279 synergizes with vincristine, paclitaxel and Alisertib

**Fig. 6:**
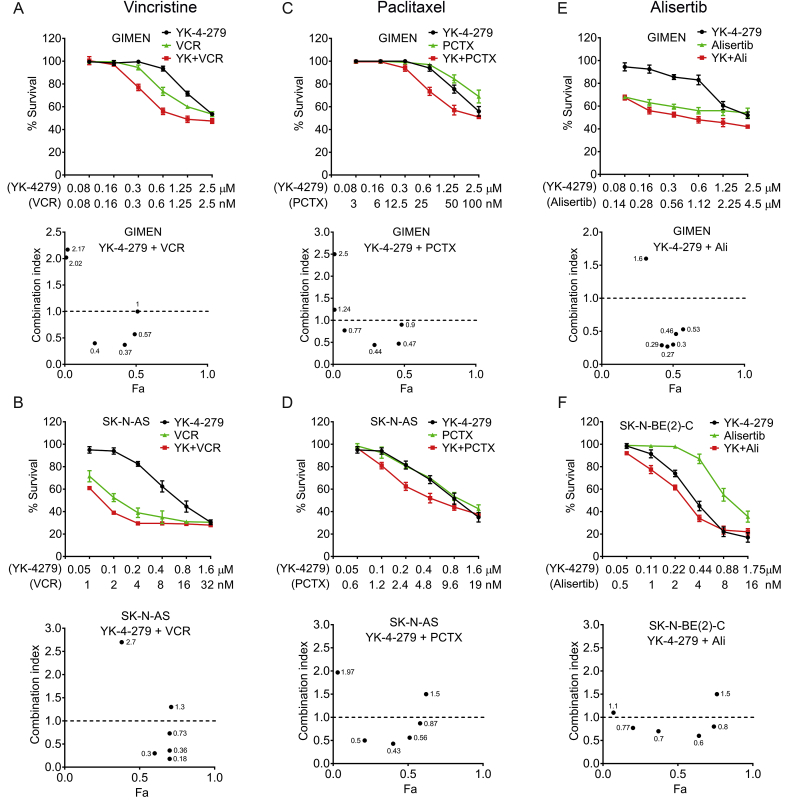
YK-4-279 synergizes with mitotic inhibitors. **(A)** GIMEN cells line were treated either singly, or with combinations of YK-4-279 and vincristine, and the MTT proliferation assay was carried out after 72 h. Dose-response curves are shown in the upper panels and Combination Index (CI) plots in the lower panels. Percentage survival values were transformed into Fraction affected (Fa) values and used to calculate combination index (measure of synergy, additivity and antagonism) using CompuSyn software. **(B)** Combination studies for SK-N-AS (YK-4-279 with Vincristine), **(C)** GIMEN (YK-4-279 with paclitaxel), **(D)** SK-N-AS (YK-4-279 with paclitaxel), **(E)** GIMEN (YK-4-279 with Alisertib) and **(F)** SK-N-BE(2)-C (YK-4-279 with Alisertib).

**Fig. S5:**
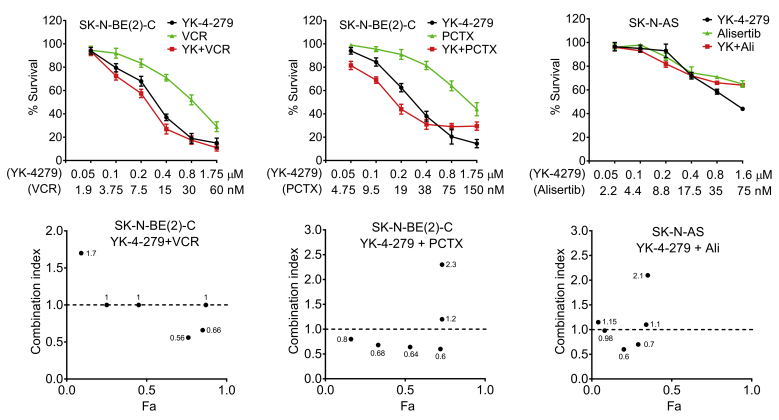
YK-4-279 effectively synergizes with mitotic inhibitors. Dose-response curves and combination index plots were generated after 72 h MTT assay for the combination studies in SK-N-BE(2)-C (YK-4-279 with vincristine or paclitaxel) and SK-N-AS (YK-4-279 with Alisertib).

Our cell cycle, cell death and live-cell imaging data show that YK-4-279 is inducing mitotic perturbations by mechanism(s) that are independent of Aurora kinase A, and different from paclitaxel and vincristine. We therefore explored whether YK-4-29 can further enhance the sensitivity to mitotic inhibitors. We determined IC_50_ values for vincristine, paclitaxel and Alisertib/MLN8237, an Aurora kinase A inhibitor in clinical trials for neuroblastoma [24], [32] and then combined the drugs in six combinational equipotent ratios based on the IC_50_ values in order to assess effects on proliferation and obtain combination indices (CI) by the method of Chou and Talaly [33]. Almost all combination dose-response curves shifted towards sensitive side of the graph indicating that combinations were more efficient in inhibiting cell proliferation. Out of six YK-4-279 and vincristine concentration combinations tested in GIMEN, three resulted in synergy with CI values 0.37, 0.4 and 0.57 (Fig. 6A). In SK-N-AS an even stronger synergy was observed (CI values: 0.18, 0.3, 0.36 and 0.73) (Fig. 6B). Only two synergistic combinations were observed in SK-N-BE(2)-C (Fig. S5). With respect to paclitaxel, three combinations resulted in synergy in GIMEN cells (CI: 0.44, 0.47, 0.77), and one close to additive effect (0.9) (Fig. 6C). Similarly, synergy was attained in SK-N-AS, with three CI values of approximately 0.5 (Fig. 6D), and in SK-N-BE(2)-C cells, particularly at lower doses (CI: 0.6, 0.64, 0.68, 0.8) (Fig. S5). Finally we also tested YK-4-279 in combination with Alisertib/MLN8237. In GIMEN cells, five out of six combinations were strongly synergistic (CI range: 0.27–0.53) (Fig. 6E), whereas SK-N-BE(2)-C cells showed four combinations with significant synergy (CI: 0.6–0.8) (Fig. 6F). In contrast, SK-N-AS cells exhibited only two combinations with synergy (CI: 0.6 and 0.7) (Fig. S5). Together these experiments highlight the potential for YK-4-279 as an adjuvant chemotherapeutic agent, enabling use of lower doses of inhibitors of mitosis in current clinical use.

## Discussion

In this study, we have shown that the small molecule inhibitor YK-4-279 causes apoptosis of neuroblastoma cell lines, irrespective of their diverse oncogenic genotypes. This inhibitor dramatically disrupts progression through mitosis by a mechanism different from the commonly used spindle poisons paclitaxel and vincristine, and is thereby able to (a) overcome cancer cell resistance to vincristine, and (b) synergize with mitotic inhibitors. Thus YK-4-279 and structurally related compounds may be highly effective for neuroblastoma therapy, either as a single agent or in combination with other drugs targeting mitosis.

Although originally reported as an agent that selectively disrupts the EWS-FLI/DHX9 interaction in Ewing's sarcoma [17], our study suggests that YK-4-279 is capable of targeting a much wider variety of cancers. Examination of YK-4-279 data available on the “Genomics of drug sensitivity of cancer” database (GDSC: http://www.cancerrxgene.org) reveals that the majority of the 821 cancer cell lines screened against YK-4-279 were sensitive, with IC_50_'s as low as 420 nM, ranging to as high as 798 μM. These data do not highlight a clear genotype underlying YK-4-279 sensitivity. Neuroblastomas do not possess the EWS-FLI fusion protein, and peptides disrupting the EWS-FLI/DHX9 interaction were reported to be ineffective against SK-N-AS cells [17]. We did not observe any changes in the levels of DHX9 following YK-4-279 treatment, or any effects on cell proliferation following DHX9 knockdown (data not shown). All neuroblastoma cell lines screened were sensitive to YK-4-279 within a narrow range of IC_50_'s, but normal fibroblasts and epithelial cells were not as sensitive to YK-4-279, consistent with the target and/or pathway(s) being more common, but nevertheless cancer-cell specific. Our demonstration that YK-4-279 sensitivity is enantiomer-specific highlights the potential of YK-4-279 as a selective therapeutic agent, despite the precise target(s) remaining unknown.

We speculate that YK-4-279 is able to disrupt key protein interactions required for controlled mitosis and proliferation of cancers cells. Our mechanistic experiments demonstrate that YK-4-279 clearly exerts its anti-cancer effects via disruption of mitosis. The mitotic defects induced by YK-4-279 lead to cells developing multipolar and fragmented spindles, as well as unseparated spindle poles, and being unable to transition from prometaphase to metaphase. Our data did not reveal obvious differential expression of mitotic regulatory proteins, or indeed the disruption of early kinase signaling events, such as Aurora A auto-phosphorylation or histone 3 phosphorylation. Entry into, and progression through, mitosis involves exquisitely sensitive and coordinated spatiotemporal signaling involving multiple protein complexes [34], and YK-4-279 may provide an excellent tool compound for future dissection of subtle mitotic phenotypes corresponding with particular proteins using the Mitocheck database (http://www.mitocheck.org/) [35]. Our microscopy studies also suggested that YK-4-279 was not interfering with tubulin and microtubule assembly, as we were clearly able to monitor tubulin with SiR-tubulin after YK-4-279 treatment, in contrast to vincristine and paclitaxel-treated cells, where SiR-tubulin binding was minimal, presumably due to competitive binding as SiR-tubulin is based on docetaxel [29]. This difference between the drugs was also apparent when assessing acetylated tubulin, which was unaffected by YK-4-279.

Importantly, we have demonstrated that this difference can be therapeutically exploited to overcome resistance to vincristine. Although vincristine is used for relapsing and refractory neuroblastoma therapy [36], resistance is apparent [31], [37] together with cross-resistance [38], [39]. We have shown that vincristine-resistant cell lines can be driven to mitotic arrest and apoptosis by YK-4-279, and also demonstrated that resistant lines which exhibit cross-resistance to several other drugs commonly used as neuroblastoma chemotherapeutics, including topotecan, doxorubicin, etoposide, cisplatin, melphalan, and busulphan, remain susceptible to YK-4-279. This underlines the possible advantages of YK-4-279 as a therapeutic agent for neuroblastoma. Interestingly, our studies also show that temozolomide, which is included in BEACON clinical trial, was the only other agent which was not susceptible to cross-resistance in our two models, thus providing a rationale for temozolomide plus vincristine combination therapies. Combinations with YK-4-279 may also be effective when the *in vivo*/clinical efficacy of YK-4-279 is verified. As well as countering resistance to spindle poisons, we further established that YK-4-279 synergized with vincristine, paclitaxel and Alisertib, especially at lower doses. These combinations may therefore achieve maximal therapeutic responses with minimal side-effects; despite its widespread usage in chemotherapy, vincristine is known to have considerable toxic side-effects [40], and our study suggests that YK-4-279 may improve the chemotherapy standard of care.

Our triple staining flow cytometry data show that most of the cell death caused by YK-4-279 is probably happening in interphase rather than there being extensive death in mitosis. Thus our data suggest that spindle assembly checkpoint [41] although induced, may permit slippage into interphase over time. Interestingly, neuroblastoma cell lines have been shown to be sensitive to depletion of Centrosome-associated protein E (CENPE), a prometaphase protein required for establishment and maintenance of kinetochores and spindle microtubule connections [42]. Unlike YK-4-279, CENPE depletion was selective for *MYCN*-amplified lines, suggesting that the target for YK-4-279 is not simply disrupting kinetochore function. However, the CENPE inhibitor GSK923295 was effective against a broad spectrum of neuroblastoma cell lines. Together with our study, this suggests that a systematic search for early mitotic vulnerabilities in neuroblastoma should be considered in the future.

Cell fate upon treatment with mitotic inhibitors depends upon the type and concentration of drug used - cells may produce aneuploid progeny or undergo mitotic slippage or undergo cell death in mitosis or interphase [43]. However, we did not see any polyploid populations in any of the cell-lines analyzed, although our analyses of GIMEN cells suggest that mitotic defects induced by YK-4-279 did activate the G1 checkpoint, as evidenced by the upregulation of functional p53 and its transcriptional target p21. Knockdown of p53 in GIMEN cells did not alleviate cell death, however, and both SK-N-AS and SK-N-BE(2)-C cells have *TP53* mutations, suggesting that apoptosis is instigated by p53-independent mechanisms. These could involve p73, which has been shown to be induced in response to DNA damaging agents and induce apoptosis [44], or MYC family proteins, which are highly expressed in neuroblastoma [45]. c-MYC was recently shown to be a key regulator of apoptosis ensuing from mitotic defects, sensitizing cancer cells to inhibitors of mitosis by transcriptional upregulation of pro-apoptotic BH3-only proteins as well as suppression of pro-survival Bcl-xL [46].

In summary, our study demonstrates that YK-4-279 is a powerful inhibitor of mitosis able to elicit apoptosis in a variety of neuroblastoma cell lines with different oncogenic drivers. It is also capable of countering drug resistance, and synergizing with inhibitors of mitosis. We are currently continuing efforts to define the target(s) of YK-4-279 and the mechanism by which apoptosis is induced. Compounds of this class likely represent an important addition to the current chemotherapy options for neuroblastoma, as well as for other cancers.

## Grant support

We would like to thank Neuroblastoma UK and Smile with Siddy (K.M.) for funding this study. Additional funding was from Cancer Research UK (A21046) (K.M.), Children with Cancer UK (K.M.), University of Bristol postgraduate scholarships (A.S. and J.H.P.), and a University of Bristol Research Fellowship (K.M.). The A.K. laboratory is further supported by the Human Frontiers Science Program (RGP0021/2016), a Medical Research Council New Investigator Award (MR/N000013/1) and Wellcome Trust Seed Award (WT107789AIA).

## References

[1] Maris J.M., Hogarty M.D., Bagatell R., Cohn S.L. (2007). Neuroblastoma. Lancet.

[2] Molenaar J.J., Koster J., Zwijnenburg D.A., van Sluis P., Valentijn L.J., van der Ploeg I. (2012). Sequencing of neuroblastoma identifies chromothripsis and defects in neuritogenesis genes. Nature.

[3] Peifer M., Hertwig F., Roels F., Dreidax D., Gartlgruber M., Menon R. (2015). Telomerase activation by genomic rearrangements in high-risk neuroblastoma. Nature.

[4] Valentijn L.J., Koster J., Zwijnenburg D.A., Hasselt N.E., van Sluis P., Volckmann R. (2015). TERT rearrangements are frequent in neuroblastoma and identify aggressive tumors. Nat. Genet..

[5] Mosse Y.P., Laudenslager M., Longo L., Cole K.A., Wood A., Attiyeh E.F. (2008). Identification of ALK as a major familial neuroblastoma predisposition gene. Nature.

[6] Ladenstein R., Potschger U., Pearson A.D., Brock P., Luksch R., Castel V. (2017). Busulfan and melphalan versus carboplatin, etoposide, and melphalan as high-dose chemotherapy for high-risk neuroblastoma (HR-NBL1/SIOPEN): an international, randomised, multi-arm, open-label, phase 3 trial. Lancet Oncol..

[7] Berry T., Luther W., Bhatnagar N., Jamin Y., Poon E., Sanda T. (2012). The ALK(F1174L) mutation potentiates the oncogenic activity of MYCN in neuroblastoma. Cancer Cell.

[8] Shukla N., Ameur N., Yilmaz I., Nafa K., Lau C.Y., Marchetti A. (2012). Oncogene mutation profiling of pediatric solid tumors reveals significant subsets of embryonal rhabdomyosarcoma and neuroblastoma with mutated genes in growth signaling pathways. Clin. Cancer Res. Off. J. Am. Assoc. Cancer Res..

[9] Eleveld T.F., Oldridge D.A., Bernard V., Koster J., Daage L.C., Diskin S.J. (2015). Relapsed neuroblastomas show frequent RAS-MAPK pathway mutations. Nat. Genet..

[10] Schramm A., Koster J., Assenov Y., Althoff K., Peifer M., Mahlow E. (2015). Mutational dynamics between primary and relapse neuroblastomas. Nat. Genet..

[11] Vieira G.C., Chockalingam S., Melegh Z., Greenhough A., Malik S., Szemes M. (2015). LGR5 regulates pro-survival MEK/ERK and proliferative Wnt/beta-catenin signalling in neuroblastoma. Oncotarget.

[12] Keld R., Guo B., Downey P., Gulmann C., Ang Y.S., Sharrocks A.D. (2010). The ERK MAP kinase-PEA3/ETV4-MMP-1 axis is operative in oesophageal adenocarcinoma. Mol. Cancer.

[13] Keld R., Guo B., Downey P., Cummins R., Gulmann C., Ang Y.S. (2011). PEA3/ETV4-related transcription factors coupled with active ERK signalling are associated with poor prognosis in gastric adenocarcinoma. Br. J. Cancer.

[14] Hollenhorst P.C., Ferris M.W., Hull M.A., Chae H., Kim S., Graves B.J. (2011). Oncogenic ETS proteins mimic activated RAS/MAPK signaling in prostate cells. Genes Dev..

[15] Lambertz I., Kumps C., Claeys S., Lindner S., Beckers A., Janssens E. (2015). Upregulation of MAPK negative feedback regulators and RET in mutant ALK neuroblastoma: implications for targeted treatment. Clin. Cancer Res. Off. J. Am. Assoc. Cancer Res..

[16] Pop M.S., Stransky N., Garvie C.W., Theurillat J.P., Hartman E.C., Lewis T.A. (2014). A small molecule that binds and inhibits the ETV1 transcription factor oncoprotein. Mol. Cancer Ther..

[17] Erkizan H.V., Kong Y., Merchant M., Schlottmann S., Barber-Rotenberg J.S., Yuan L. (2009). A small molecule blocking oncogenic protein EWS-FLI1 interaction with RNA helicase A inhibits growth of Ewing's sarcoma. Nat. Med..

[18] Rahim S., Beauchamp E.M., Kong Y., Brown M.L., Toretsky J.A., Uren A. (2011). YK-4-279 inhibits ERG and ETV1 mediated prostate cancer cell invasion. PLoS One.

[19] Michaelis M., Agha B., Rothweiler F., Loschmann N., Voges Y., Mittelbronn M. (2015). Identification of flubendazole as potential anti-neuroblastoma compound in a large cell line screen. Sci. Rep..

[20] Michaelis M., Rothweiler F., Barth S., Cinatl J., van Rikxoort M., Loschmann N. (2011). Adaptation of cancer cells from different entities to the MDM2 inhibitor nutlin-3 results in the emergence of p53-mutated multi-drug-resistant cancer cells. Cell Death Dis..

[21] Rahim S., Minas T., Hong S.H., Justvig S., Celik H., Kont Y.S. (2014). A small molecule inhibitor of ETV1, YK-4-279, prevents prostate cancer growth and metastasis in a mouse xenograft model. PLoS One.

[22] Barber-Rotenberg J.S., Selvanathan S.P., Kong Y., Erkizan H.V., Snyder T.M., Hong S.P. (2012). Single enantiomer of YK-4-279 demonstrates specificity in targeting the oncogene EWS-FLI1. Oncotarget.

[23] Nair J.S., Ho A.L., Tse A.N., Coward J., Cheema H., Ambrosini G. (2009). Aurora B kinase regulates the postmitotic endoreduplication checkpoint via phosphorylation of the retinoblastoma protein at serine 780. Mol. Biol. Cell.

[24] Gorgun G., Calabrese E., Hideshima T., Ecsedy J., Perrone G., Mani M. (2010). A novel Aurora-A kinase inhibitor MLN8237 induces cytotoxicity and cell-cycle arrest in multiple myeloma. Blood.

[25] Clute P., Pines J. (1999). Temporal and spatial control of cyclin B1 destruction in metaphase. Nat. Cell Biol..

[26] Dumontet C., Jordan M.A. (2010). Microtubule-binding agents: a dynamic field of cancer therapeutics. Nat. Rev. Drug Discov..

[27] Hammond J.W., Huang C.F., Kaech S., Jacobson C., Banker G., Verhey K.J. (2010). Posttranslational modifications of tubulin and the polarized transport of kinesin-1 in neurons. Mol. Biol. Cell.

[28] Huff L.M., Sackett D.L., Poruchynsky M.S., Fojo T. (2010). Microtubule-disrupting chemotherapeutics result in enhanced proteasome-mediated degradation and disappearance of tubulin in neural cells. Cancer Res..

[29] Lukinavicius G., Reymond L., D'Este E., Masharina A., Gottfert F., Ta H. (2014). Fluorogenic probes for live-cell imaging of the cytoskeleton. Nat. Methods.

[30] Bernard J.L., Philip T., Zucker J.M., Frappaz D., Robert A., Margueritte G. (1987). Sequential cisplatin/VM-26 and vincristine/cyclophosphamide/doxorubicin in metastatic neuroblastoma: an effective alternating non-cross-resistant regimen?. J. Clin. Oncol..

[31] Keshelava N., Seeger R.C., Reynolds C.P. (1997). Drug resistance in human neuroblastoma cell lines correlates with clinical therapy. Eur. J. Cancer.

[32] Mosse Y.P., Lipsitz E., Fox E., Teachey D.T., Maris J.M., Weigel B. (2012). Pediatric phase I trial and pharmacokinetic study of MLN8237, an investigational oral selective small-molecule inhibitor of Aurora kinase A: a Children's Oncology Group Phase I Consortium study. Clin. Cancer Res. Off. J. Am. Assoc. Cancer Res..

[33] Chou T.C., Talaly P. (1977). A simple generalized equation for the analysis of multiple inhibitions of Michaelis-Menten kinetic systems. J. Biol. Chem..

[34] Wieser S., Pines J. (2015). The biochemistry of mitosis. Cold Spring Harb. Perspect. Biol..

[35] Neumann B., Walter T., Heriche J.K., Bulkescher J., Erfle H., Conrad C. (2010). Phenotypic profiling of the human genome by time-lapse microscopy reveals cell division genes. Nature.

[36] DuBois S.G., Chesler L., Groshen S., Hawkins R., Goodarzian F., Shimada H. (2012). Phase I study of vincristine, irinotecan, and (1)(3)(1)I-metaiodobenzylguanidine for patients with relapsed or refractory neuroblastoma: a new approaches to neuroblastoma therapy trial. Clin. Cancer Res. Off. J. Am. Assoc. Cancer Res..

[37] Keshelava N., Seeger R.C., Groshen S., Reynolds C.P. (1998). Drug resistance patterns of human neuroblastoma cell lines derived from patients at different phases of therapy. Cancer Res..

[38] Keshelava N., Groshen S., Reynolds C.P. (2000). Cross-resistance of topoisomerase I and II inhibitors in neuroblastoma cell lines. Cancer Chemother. Pharmacol..

[39] Loschmann N., Michaelis M., Rothweiler F., Zehner R., Cinatl J., Voges Y. (2013). Testing of SNS-032 in a panel of human neuroblastoma cell lines with acquired resistance to a broad range of drugs. Transl. Oncol..

[40] Mora E., Smith E.M., Donohoe C., Hertz D.L. (2016). Vincristine-induced peripheral neuropathy in pediatric cancer patients. Am. J. Cancer Res..

[41] Lara-Gonzalez P., Westhorpe F.G., Taylor S.S. (2012). The spindle assembly checkpoint. Curr. Biol..

[42] Balamuth N.J., Wood A., Wang Q., Jagannathan J., Mayes P., Zhang Z. (2010). Serial transcriptome analysis and cross-species integration identifies centromere-associated protein E as a novel neuroblastoma target. Cancer Res..

[43] Topham C.H., Taylor S.S. (2013). Mitosis and apoptosis: how is the balance set?. Curr. Opin. Cell Biol..

[44] Ozaki T., Hosoda M., Miyazaki K., Hayashi S., Watanabe K., Nakagawa T. (2005). Functional implication of p73 protein stability in neuronal cell survival and death. Cancer Lett..

[45] Westermann F., Muth D., Benner A., Bauer T., Henrich K.O., Oberthuer A. (2008). Distinct transcriptional MYCN/c-MYC activities are associated with spontaneous regression or malignant progression in neuroblastomas. Genome Biol..

[46] Topham C., Tighe A., Ly P., Bennett A., Sloss O., Nelson L. (2015). MYC is a major determinant of mitotic cell fate. Cancer Cell.

