# Supplementary figures and images for: The small molecule inhibitor YK-4-279 disrupts mitotic progression of neuroblastoma cells, overcomes drug resistance and synergizes with inhibitors of mitosis
Source: Cancer Lett. 2017 Sep 10;403:74–85. doi: 10.1016/j.canlet.2017.05.027 (PMC5542135; doi:10.1016/j.canlet.2017.05.027)

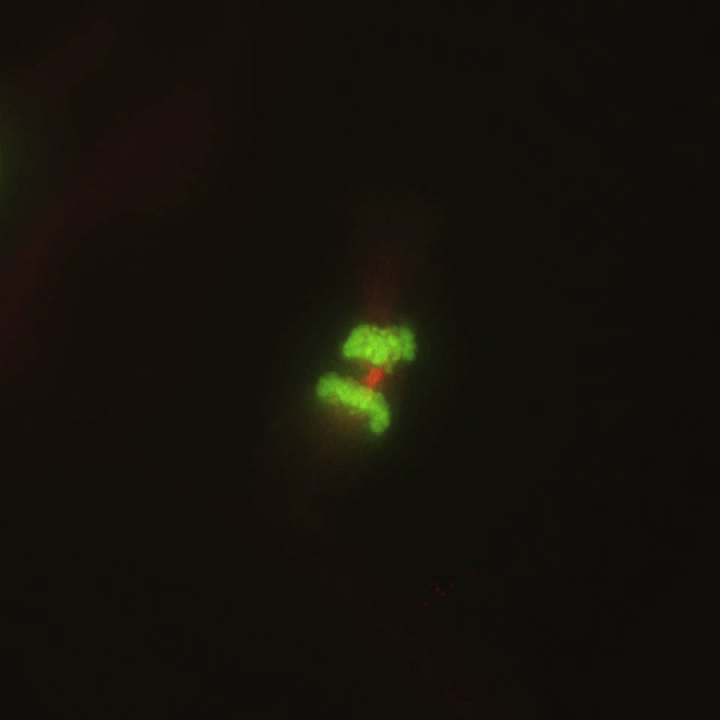

Supplement: Video 1 — GIMEN: DMSO treated. [file mmc1.jpg]

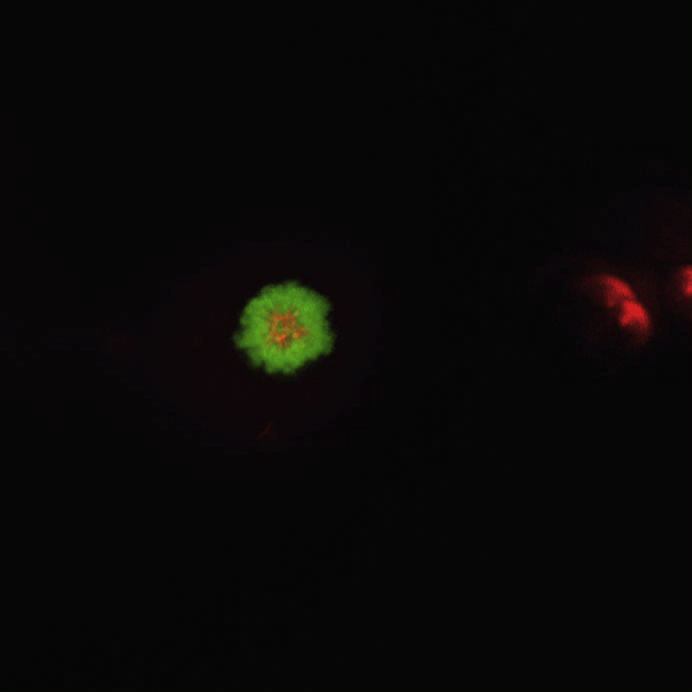

Supplement: Video 2 — GIMEN: YK-4-279 treated. [file mmc2.jpg]

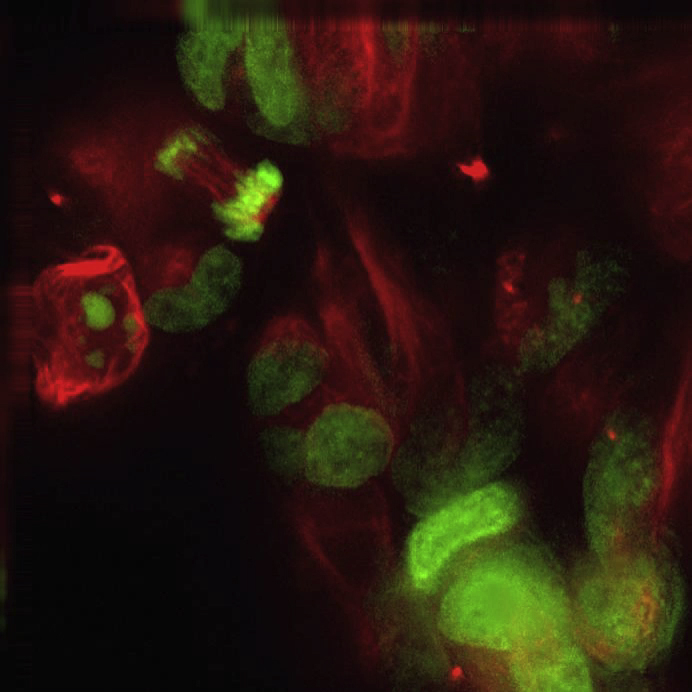

Supplement: Video 3 — SH-SY5Y: DMSO treated. [file mmc3.jpg]

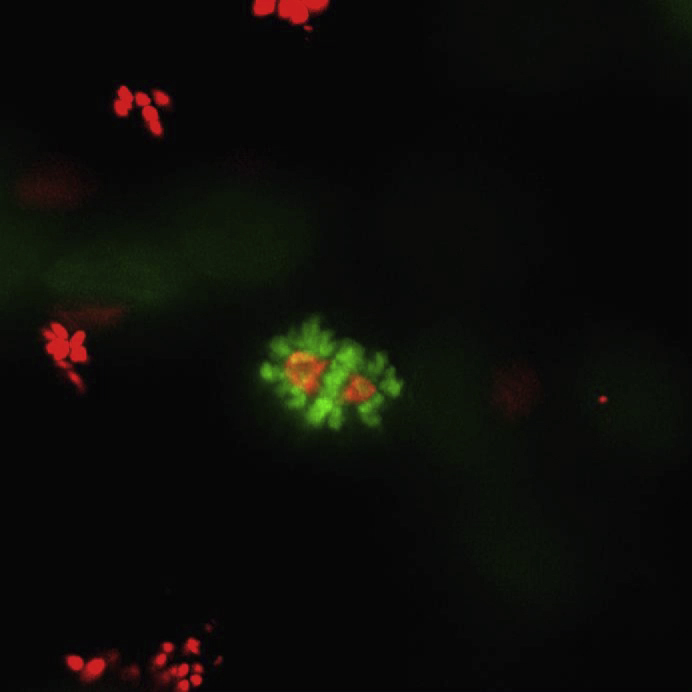

Supplement: Video 4 — SH-SY5Y: YK-4-279 treated. [file mmc4.jpg]

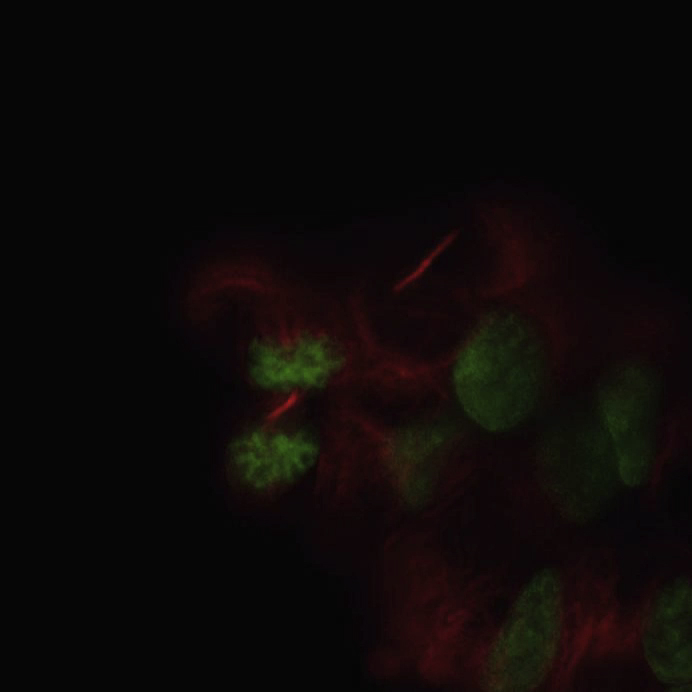

Supplement: Video 5 — SK-N-BE(2)-C: DMSO treated. [file mmc5.jpg]

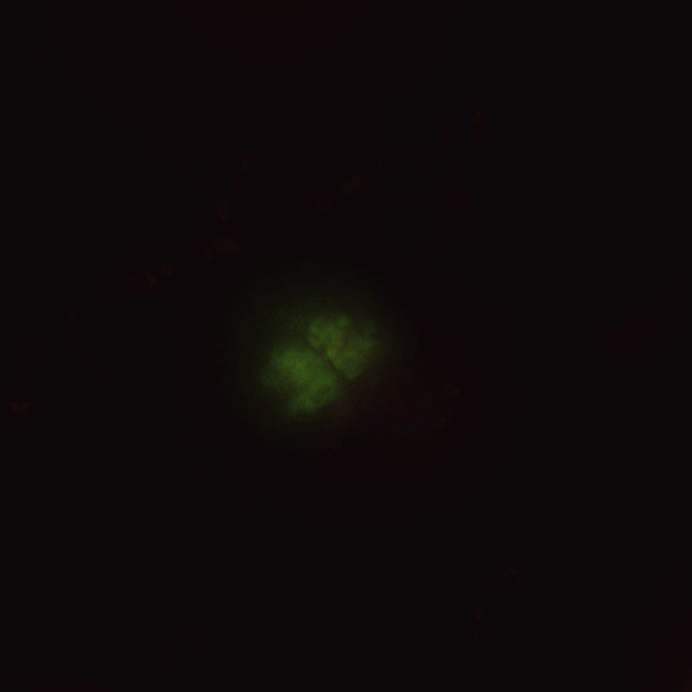

Supplement: Video 6 — SK-N-BE(2)-C: YK-4-279 treated. [file mmc6.jpg]

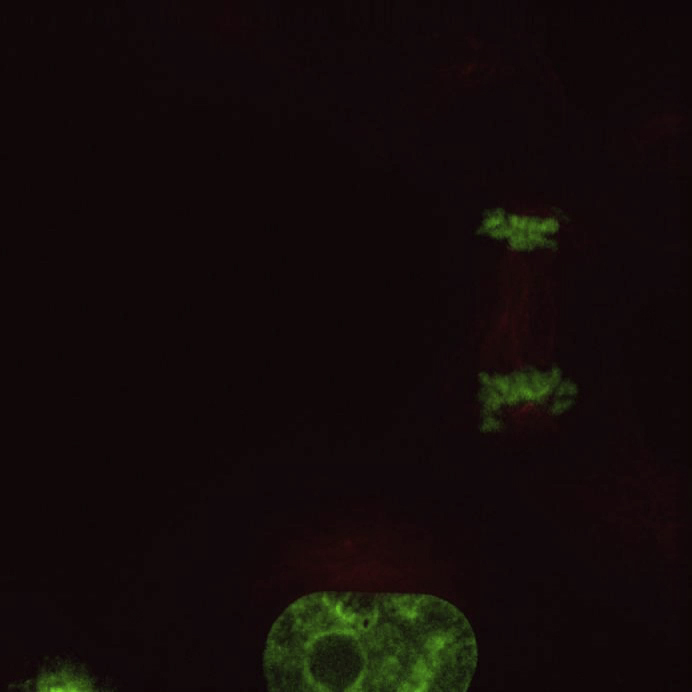

Supplement: Video 7 — SK-N-AS: DMSO treated. [file mmc7.jpg]

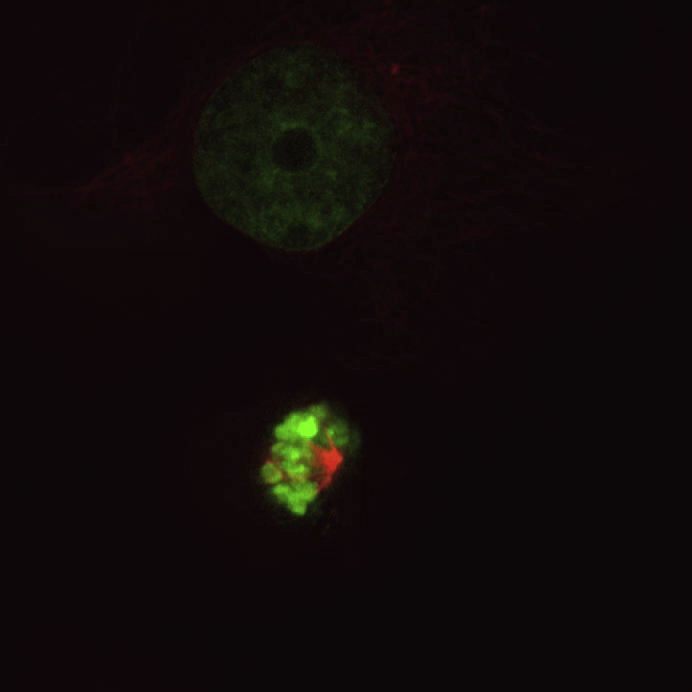

Supplement: Video 8 — SK-N-AS: YK-4-279 treated. [file mmc8.jpg]
